# Mitochondria at the Crossroads: Linking the Mediterranean Diet to Metabolic Health and Non-Pharmacological Approaches to NAFLD

**DOI:** 10.3390/nu17071214

**Published:** 2025-03-30

**Authors:** Giovanna Mercurio, Antonia Giacco, Nicla Scopigno, Michela Vigliotti, Fernando Goglia, Federica Cioffi, Elena Silvestri

**Affiliations:** Department of Science and Technology, University of Sannio, Via De Sanctis, 82100 Benevento, Italy; giomercurio@unisannio.it (G.M.); antonia.giacco@unisannio.it (A.G.); n.scopigno@studenti.unisannio.it (N.S.); mvigliotti@unisannio.it (M.V.); goglia@unisannio.it (F.G.); fecioffi@unisannio.it (F.C.)

**Keywords:** mitochondrial bioenergetics, mitochondrial quality control, polyphenols, MUFA, PUFA, fructose, fatty liver, metabolic syndrome, lifestyle, MASLD

## Abstract

Nonalcoholic fatty liver disease (NAFLD) is a growing global health concern that is closely linked to metabolic syndrome, yet no approved pharmacological treatment exists. The Mediterranean diet (MD) emerged as a first-line dietary intervention for NAFLD, offering metabolic and hepatoprotective benefits. Now conceptualized as a complex chemical matrix rich in bioactive compounds, the MD exerts antioxidant and anti-inflammatory effects, improving insulin sensitivity and lipid metabolism. Mitochondria play a central role in NAFLD pathophysiology, influencing energy metabolism, oxidative stress, and lipid homeostasis. Emerging evidence suggests that the MD’s bioactive compounds enhance mitochondrial function by modulating oxidative phosphorylation, biogenesis, and mitophagy. However, most research has focused on individual compounds rather than the MD as a whole, leaving gaps in understanding its collective impact as a complex dietary pattern. This narrative review explores how the MD and its bioactive compounds influence mitochondrial health in NAFLD, highlighting key pathways such as mitochondrial substrate control, dynamics, and energy efficiency. A literature search was conducted to identify relevant studies on the MD, mitochondria, and NAFLD. While the search was promising, our understanding remains incomplete, particularly when current knowledge is limited by the lack of mechanistic and comprehensive studies on the MD’s holistic impact. Future research integrating cutting-edge experimental approaches is needed to elucidate the intricate diet–mitochondria interactions. A deeper understanding of how the MD influences mitochondrial health in NAFLD is essential for developing precision-targeted nutritional strategies that can effectively prevent and manage the disease.

## 1. Introduction

Nonalcoholic fatty liver disease (NAFLD) is defined as a hepatic triglyceride (TG) content exceeding 5% in the absence of significant alcohol consumption or secondary causes of hepatic steatosis [1]. Representing a spectrum of liver dysfunctions—from simple steatosis to nonalcoholic steatohepatitis (NASH), fibrosis, cirrhosis, and hepatocellular carcinoma—NAFLD is closely linked to metabolic syndrome and is associated with heightened cardiovascular and metabolic risks. Its pathogenesis is multifactorial, involving a combination of genetic predisposition, dietary influences, systemic metabolic dysfunction, and organ-specific interactions. Core mechanisms at the liver level include dysregulated lipid homeostasis, mitochondrial dysfunction, inflammation, and fibrosis. Despite its growing prevalence and public health impact, no specific pharmacological therapy is currently approved for NAFLD, leaving lifestyle modifications, including weight loss, physical activity, and dietary strategies, as the cornerstone of therapeutic management [2]. Given the central role of metabolic dysfunction in NAFLD, dietary interventions are increasingly being recognized as essential tools for disease prevention and management. Indeed, specific diets may influence the key pathogenic pathways modulating disease progression.

Several dietary patterns have been explored for NAFLD management, with varying levels of evidence supporting their efficacy. Among these, the Mediterranean diet (MD), the Dietary Approaches to Stop Hypertension (DASH) diet, and low-carbohydrate diets have demonstrated promise in improving metabolic parameters and mitigating disease progression [3,4]. However, research gaps still remain. The DASH diet lacks interventional studies, and low-fat diets require further analysis, particularly regarding fat composition. Larger cohort studies are needed, along with comparisons of low-fat diets to the MD and low-carbohydrate diets. NAFLD diagnostic criteria should be improved and standardized, and data from underrepresented regions, such as Africa and South America, should be expanded. In such a scenario, only the MD has garnered consistent support from leading medical societies, including the European Association for the Study of the Liver and the American Gastroenterological Association, as a first-line dietary intervention for NAFLD prevention and management [5,6].

Since its definition in the mid-20th century, the MD has been recognized as a healthy eating pattern characterized by high intakes of whole grains, legumes, vegetables, fruits, white meat, and fish, with extra virgin olive oil (EVOO) as the principal fat source. Its composition—rich in dietary fiber, omega-3 polyunsaturated fatty acids (PUFAs), complex carbohydrates, vitamins, minerals, and bioactive phytochemicals—provides antioxidant and anti-inflammatory effects, counteracting oxidative stress and chronic low-grade inflammation. Observational and interventional studies suggest that the MD improves insulin sensitivity, lipid metabolism, and body composition, thereby preventing NAFLD and slowing down its progression [7,8].

Despite promising findings, critical questions still remain. Can the MD’s benefits be optimized under specific conditions? What are the precise molecular and biochemical mechanisms underlying its efficacy, particularly at the subcellular level? What role do organelles, such as mitochondria, play in mediating its benefits? Mitochondria, as central regulators of energy metabolism, oxidative stress, and lipid homeostasis—profoundly impacted by NAFLD and, in turn, able to influence its progression—may play a pivotal role in the MD’s potential [9]. Notably, emerging pharmacological therapies for NAFLD, such as mitochondrial-targeted antioxidants and modulators of mitochondrial biogenesis, aim to restore mitochondrial function and reduce oxidative damage, actually mimicking the effects of some of the MD’s bioactive components [10,11,12]. Understanding how the MD modulates mitochondrial bioenergetics, biogenesis, and dynamics can help identify new targets and nutraceutical interventions aimed at restoring mitochondrial integrity. This knowledge could provide crucial insights into the development of more effective, targeted strategies for mitochondrial dysfunction in NAFLD, integrating dietary approaches with pharmacological innovations if necessary. Mechanistically, a wide and heterogeneous range of data provides information on the actions of many individual, purified bioactive MD compounds, while the molecular targets of the diet, which is fundamentally a complex chemical matrix and a substance destined for digestion and processing in vivo, remain elusive. This narrative review aims to unravel these connections, providing insights into how the MD and its bioactive components influence liver metabolism and mitochondrial function while exploring their role in the management of NAFLD and its complications.

## 2. Non-Alcoholic Fatty Liver Disease (NAFLD): A Multifactorial Condition

NAFLD is the most prevalent liver disorder worldwide, affecting 25–30% of the global population. Its prevalence is significantly higher in individuals with obesity, type 2 diabetes (T2D), and metabolic syndrome. This condition, which is characterized by TG accumulation in the liver independent of substantial alcohol intake or other liver diseases, can progress to severe stages, including NASH, fibrosis, cirrhosis, and hepatocellular carcinoma (HCC). NAFLD represents a major public health challenge, due to its rising incidence and the associated burden of morbidity and mortality [13,14,15].

The pathogenesis of NAFLD is multifactorial, involving a combination of genetic predisposition, dietary influences, systemic metabolic dysfunction, and organ-specific interactions.

### 2.1. Genetic Susceptibility and Lipid Dysregulation as Primary Drivers

Human genetic analyses revealed that certain genetic variants, particularly in genes such as patatin-like phospholipase domain-containing protein-3 (PNPLA3), transmembrane 6 superfamily 2 human gene (TM6SF2), and glucokinase (hexokinase 4) regulator (GCKR), have been linked to increased susceptibility or protection from NAFLD, influencing liver fat accumulation and disease progression [16,17,18]. Studies on fatty acid transport proteins, like fatty acid transport protein 2 (FATP2) and fatty acid transport protein 5 (FATP5) and the cluster of differentiation 36 (CD36), have shown mixed findings regarding their role in hepatic fatty acid uptake. Interestingly, human studies suggest an inverse relationship between the expression of FATP5 and the severity of histologic changes in NAFLD, such as ballooning and fibrosis, suggesting the reduced role of FATP5-mediated TG accumulation as NASH progresses [19,20].

### 2.2. Dietary Factors as Key Triggers

Diets high in saturated fatty acids (SFAs), fructose, and excess calories stimulate hepatic de novo lipogenesis (DNL), a process by which acetyl-CoA is converted into palmitate, promoting TG accumulation [21,22]. Controlled-feeding studies demonstrate that fructose intake exacerbates liver fat accumulation bypassing key regulatory steps in glycolysis. Observational studies also confirm the association between habitual high fructose consumption and increased liver fat content in individuals with NAFLD [23,24].

### 2.3. Gut Microbiota Dysbiosis: A Driver of Inflammation and Disease Progression

The gut microbiota plays a crucial role in liver health, influencing metabolic homeostasis and immune regulation. In NAFLD, dysbiosis—which is characterized by alterations in microbial diversity and composition—contributes to onset and disease progression. One key factor is increased gut permeability, which allows bacterial products, such as lipopolysaccharides (LPS), to translocate to the liver via the portal vein. This process activates hepatic Toll-like receptor 4 (TLR4) signaling, promoting inflammation and fibrosis. Human studies using endotoxin assays have shown that patients with NAFLD exhibit elevated systemic LPS levels, correlating with liver inflammation and worsening histological severity [25]. Gut-derived metabolites also play a significant role in NAFLD pathophysiology. A shift of gut microbiome metabolism in NAFLD may exacerbate liver steatosis and inflammation [11]. Additionally, dysregulated bile acid metabolism impairs farnesoid X receptor (FXR) and Takeda G protein-coupled receptor 5 (TGR5) signaling, further disrupting lipid and glucose homeostasis. Recent human microbiome analyses have identified specific microbial signatures associated with NAFLD severity, reinforcing the gut–liver axis as a potential therapeutic target. Strategies aimed at restoring gut microbial balance, such as prebiotics, probiotics, and fecal microbiota transplantation (FMT), are currently being investigated for their potential to mitigate liver inflammation and metabolic dysfunction in NAFLD [26,27].

### 2.4. Obesity and Insulin Resistance: Linking NAFLD Initiation and Progression

In obesity, adipose tissue becomes inflamed and dysfunctional, releasing excess free fatty acids (FFAs) and pro-inflammatory adipokines, including tumor necrosis factor α (TNF-α) and interleukin 6 (IL-6). These factors contribute to systemic insulin resistance, which, in turn, increases hepatic lipid influx and drives excessive DNL, thereby reinforcing NAFLD development [21]. Clinical investigations reveal that patients with NAFLD exhibit significantly lower adiponectin levels, impairing lipid oxidation and enhancing inflammatory responses, thereby aggravating disease progression [28].

### 2.5. Consequences of NAFLD: Lipotoxicity, Inflammation, and Fibrosis

Once NAFLD is established, the persistence of lipid accumulation, oxidative stress, and immune activation amplifies hepatic damage. Insulin resistance worsens lipid disposal mechanisms, leading to toxic lipid intermediates that induce hepatocyte injury, mitochondrial dysfunction, and inflammation. This lipotoxic environment activates hepatic stellate cells, promoting fibrosis and setting the stage for NASH and cirrhosis [12,29].

## 3. Intrahepatic Mechanisms in NAFLD

The core mechanisms driving intrahepatic lipid accumulation in NAFLD include dysregulated lipid metabolism, impaired mitochondrial oxidation, and disrupted fatty acids (FAs) trafficking, all of which are regulated by key transcription factors and enzymes.

### 3.1. Hepatic Lipid Accumulation

Human biopsy studies consistently demonstrate that TG accumulation within hepatocytes defines NAFLD. This lipid overload results from increased FFAs, dietary lipids, and upregulated DNL, as observed in human isotopic tracer studies. DNL contributes significantly to hepatic lipid content in NAFLD patients, with studies reporting that it accounts for 26% of intrahepatic palmitate production, compared to only 10% in healthy controls [30,31]. Transcription factors such as sterol regulatory element-binding protein 1c (SREBP-1c) and carbohydrate regulatory element-binding protein (ChREBP) regulate this process. SREBP-1c, which is activated by insulin and liver X receptor alpha (LXRα), upregulates fatty acid synthase (FAS), leading to enhanced FA synthesis and liver fat accumulation [29]. In the context of NAFLD, insulin resistance upregulates SREBP-1c expression, which drives DNL and lipid accumulation. Similarly, ChREBP, which is activated by carbohydrates, stimulates glycolytic gene expression and the conversion of SFAs to monounsaturated fatty acids (MUFAs) by stearoyl-CoA desaturase 1 (SCD1), promoting TG accumulation. The elevated levels of SREBP-1c and ChREBP found in liver tissue analyses from NAFLD patients highlight their role in promoting TG accumulation and disease progression [32,33]. The accumulation of intracellular lipids, such as TGs and diacylglycerol (DAG), leads to hepatocyte lipotoxicity and the activation of pro-inflammatory pathways. Specifically, DAG activates protein kinase C epsilon (PKCε), which impairs insulin signaling and promotes insulin resistance [34,35]. This metabolic dysfunction is further exacerbated by mitochondrial dysfunction, which plays a pivotal role in NAFLD (see below). Impaired oxidative phosphorylation (OXPHOS) and mitochondrial uncoupling contribute to the accumulation of reactive oxygen species (ROS), leading to oxidative stress. ROS production damages cellular macromolecules, including lipids, proteins, and DNA, and promotes further mitochondrial dysfunction [36]. Oxidative stress is exacerbated by the reduced activity of antioxidant defense mechanisms, which is characteristic of advanced stages of NAFLD and NASH. Elevated markers of oxidative damage, including lipid peroxidation products, have been detected in plasma and liver tissue, linking oxidative stress to disease severity [37,38]. This dysfunction is particularly prominent in individuals with obesity and T2D [39,40].

### 3.2. Inflammation and Fibrosis

Inflammation is central to the progression of NAFLD, as evidenced by increased serum levels of pro-inflammatory cytokines such as TNF-α, IL-6, and IL-1β in affected individuals [41]. Liver biopsies from NAFLD patients show increased activation of the NLR family pyrin domain containing 3 (NLRP3) inflammasome and nuclear factor-kappa B (NF-κB) pathways, correlating with disease severity [42]. Longitudinal studies indicate that persistent inflammation precedes the activation of hepatic stellate cells (HSCs), which promote fibrosis. HSCs, which normally store vitamin A, become activated in response to liver injury and inflammation [43,44]. They transform into myofibroblast-like cells that produce extracellular matrix (ECM) components such as collagen, contributing to the fibrotic response. This process is driven by transforming growth factor β1 (TGF-β) and platelet-derived growth factor (PDGF), which stimulate HSC proliferation and ECM deposition. In advanced stages, excessive ECM deposition leads to fibrosis, which can progress to cirrhosis and hepatocellular carcinoma [45]. Patients with advanced fibrosis exhibit elevated circulating levels of TGF-β and PDGF, markers associated with fibrogenesis and poor outcomes [46].

### 3.3. Impaired Lipophagy

Autophagy, particularly lipophagy, plays an important role in lipid metabolism and the regulation of TG accumulation in hepatocytes. In normal conditions, lipophagy prevents excess lipid accumulation by degrading lipid droplets, but in NAFLD, impaired lipophagy exacerbates lipid storage and liver damage. Studies have shown that the inhibition of autophagy-related genes such as autophagy protein 5 (ATG5) increases hepatic lipid content and accelerates liver injury, indicating that enhancing autophagic activity may be a therapeutic strategy to mitigate NAFLD progression [47].

### 3.4. Lipid Trafficking

Finally, very-low-density lipoprotein (VLDL) export, the process by which TG is secreted from hepatocytes into the bloodstream in the form of VLDL particles, plays a role in lipid disposal. However, VLDL secretion becomes compromised in NASH when the hepatic TG content exceeds a certain threshold [48]. The formation of VLDL particles occurs in the endoplasmic reticulum (ER) through the lipidation of apolipoprotein B100 (apoB100), catalyzed by microsomal triglyceride transfer protein (MTTP). Evidence indicates that apoB100 synthesis rates and hepatic MTTP levels are lower in humans with NASH compared to those without NASH, contributing to impaired lipid export [49,50].

Preclinical studies in animal models and cellular systems have provided complementary insights into the pathogenesis of NAFLD. For example, murine models of obesity and NAFLD, such as those involving high-fat diet-based feeding or the genetic manipulation of PNPLA3 [51] and ATG5 [52], have revealed key mechanisms driving lipid accumulation and mitochondrial dysfunction. Similarly, in vitro studies using hepatocyte cultures have shown how impaired mitochondrial activity and lipotoxicity promote ROS production and hepatocyte injury [53].

These findings underscore the translational potential of targeting these pathways for therapeutic intervention. Indeed, restoring autophagy and modulating inflammatory responses offer new potential therapeutic avenues for the management of NAFLD and its progression to more severe liver diseases, such as NASH and cirrhosis. Currently, growing research interest is being devoted to improving mitochondrial function.

## 4. Mitochondrial Dysfunction in NAFLD: Bioenergetics, Biogenesis, and Dynamics

Mitochondrial dysfunction is a pivotal factor in the pathogenesis of NAFLD, contributing to metabolic, structural, and molecular disruption in liver cells. This dysfunction, evident in even the early stages of NAFLD, involves alterations in mitochondrial bioenergetics, biogenesis, and dynamics, as observed in both human and experimental model studies [54].

### 4.1. Bioenergetics: Alterations in β-Oxidation and ROS Production

In early NAFLD, hepatic mitochondria show increased β-oxidation rates, an adaptive response to the influx of FFAs [47]. Studies using high-resolution respirometry in liver tissue from obese and lean individuals have demonstrated enhanced OXPHOS capacity in non-steatotic obese livers, compared to lean controls [37]. This suggests mitochondrial plasticity as a protective mechanism against lipid overload [55]. However, in steatotic obesity, these adaptive mechanisms may fail, as reflected in inconsistent findings on ATP content and mitochondrial function [56]. In experimental models, increased β-oxidation has been linked to the excessive production of ROS, leading to mitochondrial damage. Elevated ROS levels, as observed in animal models of steatosis, cause oxidative stress and impair mitochondrial membrane integrity. Over time, these disruptions lead to electron transport chain dysfunction, ATP depletion, and lipid peroxidation, contributing to the progression from simple steatosis to NASH [57,58]. Experimental models have also highlighted the role of cytochrome P450 2E1 (CYP2E1) in mitochondrial ROS production and oxidative stress during NAFLD progression. Increased CYP2E1 activity, as observed in animal models of NASH, further amplifies oxidative damage, creating a feedback loop that worsens mitochondrial dysfunction [59,60].

### 4.2. Biogenesis: Adaptive Responses

In individuals with non-steatotic obesity, mitochondrial biogenesis appears to be upregulated, being potentially mediated by peroxisome proliferator-activated receptor gamma coactivator 1-alpha (PGC-1α) activation. However, studies on isolated liver mitochondria have reported reduced expression of the genes involved in mitochondrial biogenesis in steatotic obesity. This highlights a transition from an adaptive phase in early NAFLD to impaired mitochondrial function in advanced stages [61]. Animal studies confirm that mitochondrial biogenesis is enhanced in early NAFLD as an adaptive response to lipid overload [62]. Over time, however, this process becomes insufficient, with suppressed biogenesis being observed in later stages. Experimental findings show reduced mitochondrial DNA (mt-DNA) content [63] and altered expression of key regulators, such as PGC-1α, thereby contributing to the progression of mitochondrial dysfunction [64].

### 4.3. Dynamics and Structural Integrity

Data on mitochondrial dynamics in NAFLD are limited, but the findings suggest significant alterations in fission and fusion processes, which are critical for maintaining mitochondrial function. Observations from liver biopsies indicate disrupted mitochondrial morphology and swelling, particularly in steatotic and NASH livers [65]. Animal studies have provided more detailed insights into mitochondrial dynamics. The disruption of fusion proteins (e.g., mitochondrial fusion protein mitofusin-2 (MFN2)) [66] and the excessive activation of fission proteins (e.g., dynamin-related protein 1 (DRP1)) [67] have been implicated in mitochondrial fragmentation. These changes impair mitochondrial network integrity, exacerbate ROS production, and contribute to hepatocellular damage [54].

### 4.4. Mitophagy and Mitochondrial Quality Control

Mitophagy, the selective autophagic elimination of damaged mitochondria, plays a crucial role in maintaining mitochondrial health and preventing the progression of NAFLD. It involves both ubiquitin-dependent and -independent pathways, where the PTEN-induced kinase 1 (PINK1)/Parkin pathway is the most widely studied mechanism [68]. In dysfunctional mitochondria, PINK1 stabilizes and recruits Parkin, which ubiquitinates outer mitochondrial membrane (OMM) proteins. This process facilitates the recruitment of autophagic adapters like ubiquitin-binding protein p62 (p62), the neighbor of BRCA1 gene 1 (NBR1), and histone deacetylase 6 (HDAC6), which interact with microtubule-associated protein 1A/1B-light chain 3 (LC3) to ensure the sequestration of impaired mitochondria into autophagosomes. Additionally, receptor-mediated mitophagy pathways, involving proteins such as the BCL2/adenovirus E1B 19 kDa protein-interacting protein 3 (BNIP3) and NIX, also contribute to mitochondrial clearance. Studies in animal models show that Bnip3 gene deletion increases ROS levels and inflammation, underscoring the importance of these pathways in mitochondrial quality maintenance [68,69,70]. In human studies, mitophagy dysfunction has been linked to the severity of NAFLD [71]. Emerging evidence suggests that metabolic stressors such as insulin resistance, lipotoxicity, and hypoxia impair mitophagy through multiple mechanisms, including reduced PINK1 stabilization, impaired Parkin translocation, and excessive mitochondrial fragmentation, all of which contribute to mitochondrial dysfunction and exacerbate NAFLD progression [54]. Furthermore, disruptions in Parkin-mediated mitophagy, which is regulated by pathways such as 5′ AMP-activated protein kinase (AMPK), have been implicated in hepatocyte apoptosis and mitochondrial dysfunction [72]. AMPK activation enhances mitophagy by phosphorylating Unc-51-like autophagy-activating kinase 1 (ULK1), a key initiator of autophagy, while its impairment in NAFLD leads to reduced mitochondrial turnover and excessive ROS accumulation. Additionally, transcription factors such as transcription factor EB (TFEB) and PGC-1α have emerged as key regulators of mitophagy. TFEB, a master regulator of lysosomal function, promotes mitophagy gene expression, while PGC-1α enhances mitochondrial turnover by coordinating mitophagy with mitochondrial biogenesis. In NAFLD, these pathways are often dysregulated, impairing mitochondrial adaptation to metabolic stress [68,71]. Overall, mitochondrial quality control mechanisms (MQC) are being considered more and more essential for preserving mitochondrial integrity and functionality in the face of metabolic stress. Excessive lipid accumulation in hepatocytes triggers increased FA oxidation and ROS production, creating a cycle of mitochondrial damage [73,74]. MQC employs a multi-layered response: initial repair mechanisms include mt-DNA repair, antioxidant defense, and protein folding. When these fail, mitochondrial dynamics help isolate and repair mildly damaged mitochondria or segregate severely damaged ones for elimination via mitophagy [75,76]. Recent studies highlight the role of NAD-dependent deacetylase sirtuins (SIRT1 and SIRT3) in modulating mitophagy and MQC. SIRT1 deacetylates key autophagy-related proteins, enhancing mitophagy, while SIRT3 regulates mitochondrial protein acetylation, promoting antioxidant defense and mitochondrial adaptation. In NAFLD, SIRT1/SIRT3 downregulation is associated with defective mitophagy and increased oxidative damage, accelerating disease progression [54]. Notably, endothelial nitric oxide synthase (eNOS) has been identified as a master regulator of MQC. Studies in eNOS-deficient mice demonstrate an increased susceptibility to liver inflammation and fibrosis, due to compromised mitochondrial function [77]. The failure of mitophagy and MQC not only amplifies mitochondrial dysfunction and ROS production but also contributes to hepatic inflammation and fibrogenesis, reinforcing the transition from simple steatosis to NASH and fibrosis. The loss of MQC efficiency is now considered a key factor in the progression of NAFLD, highlighting its potential as a therapeutic target.

Given the pivotal role of mitochondria in NAFLD progression, strategies to restore mitochondrial function are being actively explored. In the absence of established pharmacological treatments, dietary interventions are increasingly being recognized as “mitochondrial nutritional approaches”. While certain dietary patterns and excess nutrients, such as high fructose intake and saturated fats, can negatively impact mitochondrial function—promoting oxidative stress and metabolic dysregulation—specific nutrients and bioactive compounds can positively modulate mitochondrial bioenergetics, dynamics, and quality control, offering promising strategies to prevent and mitigate NAFLD. However, despite significant research efforts, we are only just beginning to unravel the intricate relationship between diet and mitochondria. For further discussion, see the next section.

## 5. NAFLD Treatment Options: From Pharmacological Advances to Lifestyle Interventions

### 5.1. Pharmacological Approaches

Treatment options for NAFLD remain limited, with pharmacological approaches offering only partial efficacy [54]. Among the few approved therapies, Vitamin E has shown promise in non-diabetic patients with NASH due to its antioxidant properties, although concerns regarding its long-term safety persist [1,78]. The PIVENS trial, a landmark study involving 247 adults with biopsy-proven NASH but without diabetes, demonstrated that Vitamin E (800 IU/day) significantly improved NASH resolution compared to the placebo (43% vs. 19%, *p* = 0.001), although it did not improve fibrosis [78]. Notably, the trial also evaluated Pioglitazone, a proliferator-activated receptor gamma (PPAR-γ) agonist (30 mg/day), which caused improvements in liver histology but was associated with significant weight gain (+4.7 kg over 96 weeks). Moreover, potential side effects such as increased bleeding risk, prostate cancer, and heart failure necessitate careful patient selection [79]. Emerging treatments, such as glucagon-like peptide 1 (GLP-1) receptor agonists (e.g., liraglutide and semaglutide) and sodium-glucose transporter protein 2 inhibitors (SGLT2i), offer metabolic benefits and potential liver improvements, although further validation in large-scale trials is needed [80,81]. Other agents, including FXR agonists like obeticholic acid, have caused fibrosis reduction but remain limited by side effects [82,83].

### 5.2. Targeting Mitochondrial Dysfunction

Given the key role of mitochondrial dysfunction in NAFLD pathogenesis, targeting mitochondrial health has gained attention. Mitochondria-targeted antioxidants, such as Mito-Q and MitoVit-E, have demonstrated efficacy in reducing oxidative stress and preserving mitochondrial integrity, thereby improving hepatic lipid metabolism [84,85]. NAD-dependent deacetylase sirtuin-3 (SIRT3) activators, known for enhancing β-oxidation, reducing ROS, and promoting mitochondrial biogenesis, have shown promise in mitigating NAFLD progression [86,87]. Additionally, mitotherapy, an innovative approach involving the transplantation of functional mitochondria that are administered intravenously, has shown potential in preclinical models by restoring normal hepatic metabolism and improving cellular redox balance [88,89]. Antidiabetic drugs, such as pioglitazone and liraglutide, also exert beneficial effects on mitochondrial dynamics by enhancing β-oxidation and maintaining mitochondrial integrity [90,91]. Moreover, SGLT2 inhibitors have been implicated in reducing oxidative stress and modulating mitochondrial activity, although further research is necessary to establish their histological benefits in NAFLD patients. Hormonal regulation, particularly thyroid hormones, has also been shown to enhance mitochondrial biogenesis, β-oxidation, and mitophagy by modulating the expression of key autophagy-related genes like NIX, BNIP3, and LC3 [92]. This indicates that hormonal interventions may hold therapeutic potential for restoring mitophagy and mitigating NAFLD progression [93,94].

Unfortunately, despite all the promising preclinical findings discussed above, significant gaps remain in translational research and human clinical trials. Challenges such as bioavailability, targeted mitochondrial delivery, and potential off-target effects limit the clinical applicability of these interventions. While mitotherapy, mitochondrial-targeted antioxidants, and SIRT3 activators show therapeutic potential, issues related to mitochondrial engraftment efficiency, metabolic integration, and long-term safety remain unresolved. Similarly, pharmacological approaches, including antidiabetic and hormonal treatments, offer indirect mitochondrial benefits but lack specificity in terms of targeting hepatic mitochondria. Further research is needed to refine these strategies, optimize their therapeutic window, and assess their long-term effects on NAFLD patients. Large-scale, well-controlled clinical trials are crucial for establishing their safety, efficacy, and histological benefits before the translation of such interventions into clinical practice.

### 5.3. Surgical Interventions

For patients with severe obesity and advanced disease, bariatric surgery remains a viable option, leading to significant histological improvements in steatosis, inflammation, and fibrosis. A meta-analysis reported resolution rates of 66% for steatosis and 40% for fibrosis, although the transient worsening of NAFLD has been observed due to increased lipolysis post-surgery [95,96].

### 5.4. Lifestyle and Dietary Interventions

Despite the advancements discussed above, both pharmacological and surgical interventions for NAFLD remain limited by significant challenges. Pharmacological treatments often come with side effects, a lack of long-term safety data, and variability in patient response, while bariatric surgery, although effective, is an invasive approach with potential post-surgical complications. Given these limitations, lifestyle interventions, despite their own challenges—such as patient adherence, sustainability, and the need for personalized approaches—remain the cornerstone of NAFLD management and offer significant benefits. Sustained weight loss, achieved through a combination of dietary modification and physical activity, significantly reduces intrahepatic fat, improves insulin sensitivity, and enhances mitochondrial function [97,98]. Regular physical activity, which promotes mitochondrial biogenesis and redox balance, further mitigates disease progression [99,100]. Dietary strategies, particularly adherence to the MD, have shown metabolic and hepatic benefits [3,4]. The MD, which is rich in unprocessed foods, polyphenols, and anti-inflammatory components, has been linked to improved intrahepatic lipid levels, reduced fibrosis, and better metabolic outcomes in NAFLD patients [7,101]. While current guidelines support dietary changes, including MD-based patterns, further high-quality studies are needed to define the optimal nutritional interventions [5,102].

To understand the hepatic protective effects of the MD, it is important to clarify its general formulation and its supply of bioactive chemical substances, as discussed below.

## 6. The Mediterranean Diet: A Complex Chemical Matrix, Rich in Natural Bioactive Compounds

The concept of the MD emerged in the mid-20th century as a result of the “Seven Countries Study” led by Ancel Keys between 1958 and 1964. This landmark study compared the dietary habits of various populations across the United States (EE. UU.), Japan, Finland, the Netherlands, the former Yugoslavia, Italy, and Greece. The follow-up studies on these cohorts over a period of 5–15 years revealed that populations in Mediterranean countries, particularly Greece, exhibited lower mortality rates from coronary and general diseases, as well as greater life expectancy [103]. This comprehensive analysis of the study enabled researchers to identify specific dietary components associated with increased life expectancy, a reduced incidence of chronic diseases, and cardiovascular benefits and led Keys to coin the term “Mediterranean diet” to describe this form of nutrition [104].

It was later recognized that the MD is not a singular dietary model, as the specific foods included vary among Mediterranean regions. Nevertheless, diets from European countries such as Spain, France, Italy, and Greece, as well as those from North Africa and the Middle East, share certain core characteristics, including: low consumption of red meat, butter, and whole milk, a source of SFAs; intake of extra virgin olive oil (EVOO), a primary source of MUFAs; consumption of fish, shellfish, and nuts, which are important to furnish a balanced ratio of PUFAs (omega-6 to omega-3); limited intake of animal-derived proteins, particularly from red meat; abundant consumption of plant-based foods such as whole grains, vegetables, legumes, fruits, nuts, EVOO, and herbs, which are rich in naturally occurring antioxidants and are a precious source of dietary fiber [105].

This dietary schema is now represented by the MD pyramid, a practical guide for selecting foods based on both their quality and quantity, outlining the relative proportions and recommended frequencies for consuming the main food groups. The emphasis on variety, culinary techniques, and appropriate portion sizes determines whether the diet is health-promoting. Specifically, MD, incorporating a wide range of foods, reduces the risk of nutrient deficiencies. Accordingly, a typical MD-based balanced composition of main meals should include wholegrain cereals, vegetables, and fruits, complemented by moderate amounts of other plant-based foods, dairy products, and protein sources to contribute to daily energy needs, with a macronutrient distribution of approximately 35% fats, 15% proteins, and 50% carbohydrates, to aligns with recommendations for metabolic health [106].

### 6.1. Wholegrain Cereals

A cornerstone of the MD is the consumption of whole grains, a tradition rooted in the rich biodiversity of Mediterranean agriculture. Cereals (2.8–3.6 Kcal/g) such as *Avena sativa* (oats), *Triticum* species (wheat), *Zea mays* (corn), *Panicum miliaceum* (millet), *Oryza sativa* (rice), and *Hordeum vulgare* (barley) have long been staple foods in Mediterranean civilizations, providing carbohydrates, dietary fiber, and bioactive molecules that are now known to show antioxidant, anti-cancer, and anti-thrombotic properties [107,108]. Cereals and their derivatives, such as bread and pasta, contribute about 50% of the total caloric intake of the MD and are, therefore, placed at the base of its food pyramid [109]. Unlike refined cereals, whole grains retain the bran and germ, which contain numerous bioactive compounds that support health and help mitigate metabolic disorders [110,111].

Of these, wheat is an essential source of starch, dietary fiber, and energy. It also provides significant amounts of protein (10–12%, 60–70% of which is gluten), B vitamins [B1 (thiamin), B2 (riboflavin), B3 (niacin), B6 (pyridoxine) and B9 (folate), carotenoids, polyphenols (phenolic acids and lignans) benzoxazinoids, vitamin E, alkyl-resorcinol, phytosterols, and biogenic amines [109]. The high fiber content in whole wheat (8–10%) promotes digestion and supports regular bowel movements, potentially reducing the risk of intestinal cancer [112]. Furthermore, its phenolic compounds, vitamin E, carotenoids, and terpenoids exhibit antioxidant properties, scavenging free radicals and protecting against oxidative damage [111,113].

Dose-response meta-analyses of prospective observational studies have shown that a high consumption of whole grains is associated with a reduced risk of all-cause mortality, cardiovascular disease (CVD), T2D, colorectal cancer (CRC), hypertension, and obesity [114]. Meta-analyses of RCTs further demonstrate the beneficial effects of whole grain intake on total cholesterol, low-density lipoprotein cholesterol (LDL-C), TGs, and fasting glucose [115].

### 6.2. Vegetables and Fruit

Vegetables, fruit, and tea have a special place in the MD as low-energy, low-fat, low-sodium, cholesterol-free foods and are a precious source of fiber, complex carbohydrates, vitamins, minerals, and polyphenols, which together elicit antioxidant, anti-genotoxic, anti-cancer, and anti-inflammatory actions [116]. The Mediterranean region’s agricultural heritage includes a diverse array of plant families: Liliaceae (onions, garlic, and leeks), Solanaceae (tomatoes, peppers, and eggplants), Chenopodiaceae (spinach and beets), Asteraceae (lettuce and artichokes), Brassicaceae (cabbage, broccoli, and cauliflower), Cucurbitaceae (zucchini, cucumbers, and melons), and Apiaceae/Umbelliferae (carrots, fennel, and parsley), which are all deeply embedded in Mediterranean culinary traditions. The emphasis is on seasonal, field-grown products, and attention is paid to cooking methods that can greatly influence the chemical composition, bioactivity, and availability of the characterizing compounds [117,118,119].

Vegetables such as tomatoes (≃0.23 Kcal/g), eggplant (≃0.23 Kcal/g), cucumber (≃0.16 Kcal/g), cabbage (0.20–0.32 Kcal/g), arugula (≃0.30 Kcal), radishes (≃0.13 Kcal), garlic (≃0.50 Kcal/g), onions (≃0.28 Kcal/g), spinach (≃0.35 Kcal/g), and lettuce (≃0.22 Kcal/g) are key sources of flavonoids in the MD, together with fiber (0.1–3%), potassium, magnesium, iron, copper, and vitamins (A, E, C, K, and B group) [120].

The Mediterranean region’s biodiversity is also reflected in its traditional fruit-bearing plants, spanning several botanical families. These include the Ampelidaceae (grapes), Cactaceae (prickly pears), Caesalpiniaceae (carob), Cucurbitaceae (melons), Ebenaceae (persimmons), Ericaceae (strawberries), Grossulariaceae (currants, gooseberries), Moraceae (figs, mulberries), Platanaceae (sycamore figs), Oleaceae (olives), Arecaceae/Palmae (dates), Rosaceae (apples, pears, cherries, apricots, and peaches), Rutaceae (citrus fruits), and Rhamnaceae (jujube).

MD fruits such as citrus fruits (0.15–0.85 Kcal/g), pomegranates (≃0.68 Kcal/g), berries (≃0.42 Kcal/g), figs (≃0.63 Kcal/g), grapes (≃0.64 Kcal/g), and “orange fruits” (e.g., apricots, peaches, nectarines, and cantaloupes) (0.28–0.45 Kcal/g) are all rich in fiber (1–8%), potassium, and vitamin C but are also high in flavonoids and terpenes [120].

Several studies have highlighted the beneficial effects of a higher consumption of vegetables, fruits, and tomatoes [114,115,121,122,123,124]. Dietary consumption of ripe red tomatoes (rich in polyphenols, such as flavanones and flavones, and carotenoids, such as phytoene, β-carotene, and lycopene) has been associated with the inhibition of the onset and the progression of chronic diseases [125]. Tomatoes’ polyphenols and carotenoids modulate LDL-C and the corresponding atherogenic processes in endothelial cells and reduce the risk of CVD and atherosclerosis [126]. Lycopene is one of the most widely studied carotenoids found in tomatoes [127].

### 6.3. Legumes

Legumes (2.80–3.30 Kcal/g for dried legumes, 0.64–1.5 Kcal/g for fresh legumes) represent a fundamental food category in the MD and are consumed regularly within this nutritional framework [128]. Mediterranean agricultural traditions include a variety of leguminous species, each playing a role in both nutrition and sustainable farming practices. Key legumes include *Cicer arietinum* (chickpeas), *Lathyrus sativus* (grass peas), *Phaseolus vulgaris* (common beans), *Vicia faba* (fava beans), *Ervum lens* (lentils), *Lupinus albus* (white lupins), *Pisum sativum* (peas), and *Glycine max* (soybeans).

Over time, human societies have recognized the numerous nutritional benefits of legumes, which are rich in proteins (20–25% for dried legumes and 5–11% for fresh legumes), starch, fiber (11–17% for dried legumes and 4–7% for fresh legumes), B vitamins, and essential minerals (iron, zinc, calcium, potassium, phosphorus, magnesium, sodium, folic acid, etc.), making them a valuable component of a balanced diet [120,129]. Within the traditional MD, common legumes such as beans, chickpeas, lentils, and peas are prepared in a variety of ways, including soups, salads, stews, and main dishes [130], either on their own or in combination with other fresh foods. They provide an important source of vegetable protein—2–3 times higher than that of wheat and rice [131]—offering a sustainable and cost-effective alternative to animal protein [132,133]. The fiber content in legumes is considered vital for maintaining digestive health, as well as for regulating weight and blood sugar levels [134]. Furthermore, their low SFA content, along with the presence of PUFAs and MUFAs, antioxidants, vitamins, and minerals, contributes to overall heart and general health [135]. Legumes are also abundant in phytosterols (sterols and stanols) and isoflavones. Phytosterols have been shown to possess antioxidant, antipyretic, anti-inflammatory, and hormone-like properties, as well as anticancer, antidiabetic, antiatherosclerotic, and neuroactive effects [136]. Specifically, they demonstrate beneficial effects on lipid metabolism and contribute to the prevention and progression of NAFLD [137].

The positive effects of legume intake on various health parameters have been highlighted by several meta-analyses [115,121,122].

### 6.4. Fish/Seafood

Seafood (0.5–2.6 Kcal/g) is another key component of the MD. The Mediterranean Sea has historically offered a diverse array of marine resources, including species from different taxonomic groups, such as *Sardina pilchardus* (sardines), *Engraulis encrasicolus* (anchovy), *Thunnus thynnus* (bluefin tuna), *Dicentrarchus labrax* (sea bass), and *Sparus aurata* (gilt-head bream); cephalopods like *Octopus vulgaris* (octopus) and *Loligo vulgaris* (squid); crustaceans such as *Penaeus kerathurus* (Mediterranean prawn); and mollusks including *Mytilus galloprovincialis* (mussels) and *Ostrea edulis* (oysters). This food group provides a unique array of nutrients with significant metabolic and hormonal importance. Indeed, seafood (SFAs 25–45%, MUFAs 20–50%, and PUFAs 25–40%) is not only a source of high-quality proteins—generally offered in a context of relatively low caloric density—but also supplies omega-3 PUFAs, iodine, selenium, and vitamins (A, B, D, and K), as well as carnitine.

Regular fish consumption has been associated with numerous health benefits, impacting conditions such as metabolic syndrome, obesity, diabetes, hypothyroidism, and other hormonal disorders [138]. Amino acids derived from fish proteins have demonstrated protective effects, particularly on blood pressure and lipid profiles [139]. Lysine, an essential amino acid that is scarce in cereals, is abundantly present in fish, alongside carnitine—a crucial compound that facilitates the transport of FAs into the mitochondrial matrix for oxidation. This role is central to energy metabolism, especially in skeletal muscle, where regular fish consumption has shown the potential to preserve or improve muscle mass [140].

Iodine and selenium, which are often of limited availability in typical diets, are essential for thyroid hormone synthesis and activity [141]. Iodine plays a fundamental role as a structural component of thyroid hormones (thyroxine (T4) and triiodothyronine (T3)), which regulate energy metabolism, thermogenesis, and lipid oxidation [142]. A deficiency in dietary iodine can impair thyroid function, leading to hypothyroidism and metabolic dysregulation, with potential consequences such as weight gain, insulin resistance, and dyslipidemia [143]. Given that the iodine content in plant-based foods depends on soil composition and varies widely across regions, seafood serves as one of the most reliable dietary sources of this micronutrient. Selenium acts as a cofactor for selenoproteins, including glutathione peroxidases and thioredoxin reductases, which protect against oxidative stress and regulate immune function. Importantly, selenium is required for the activity of iodothyronine deiodinases, the enzymes responsible for converting T4 into its biologically active form, T3. This step is crucial for maintaining adequate thyroid hormone signaling and metabolic homeostasis. Selenium deficiency has been linked to impaired thyroid function, an increased risk of autoimmune thyroid disorders, and altered lipid metabolism [144]. In the context of NAFLD, selenium’s antioxidant properties may help mitigate mitochondrial oxidative stress and inflammation, thereby reducing liver damage [145]. Seafood sources rich in selenium, such as tuna, sardines, and shellfish, can contribute to maintaining adequate levels of this micronutrient and supporting both thyroid and metabolic health.

However, the most extensively studied components of fish are PUFAs, particularly omega-3 PUFAs, such as eicosapentaenoic acid (EPA 7–20%) and docosahexaenoic acid (DHA 5–22%). These FAs are abundant in oily fish but are rare among terrestrial organisms and are considered conditionally essential nutrients [120]. Their essentiality stems from their roles in maintaining cell membrane integrity and facilitating hormonal receptor signaling [146]. EPA and DHA have demonstrated broad benefits in terms of cardiovascular health, lipid profile improvement, inflammation reduction, insulin resistance mitigation, neurocognitive disorder prevention, and cancer risk reduction [147,148]. Clinical trials have also explored the effects of omega-3 PUFAs on NAFLD and NASH. A systematic review and meta-analysis of controlled intervention studies found that omega-3 supplementation significantly reduces liver fat content and steatosis scores in NAFLD patients [149]. However, its effects on the markers of severe liver injury, such as inflammation and fibrosis, remain inconclusive. Notably, adherence to the MD has been linked to higher tissue levels of omega-3 PUFAs [150].

Meta-analyses of prospective observational studies and RCTs indicate that high fish intake positively influences HDL cholesterol and TGs [114,115,121,122,151]. These benefits are thought to stem from mechanisms such as reduced inflammation, oxidative stress, and coagulation [152].

### 6.5. Nuts

The Mediterranean region is rich in diverse nut varieties, including *Corylus avellana* (hazelnuts), *Juglans regia* (walnuts), *Prunus dulcis* (almonds), *Pistacia vera* (pistachios), and *Castanea sativa* (chestnuts). All nuts (almonds (≃6.28 Kcal/g), Brazil nuts (≃5.90 Kcal/g), cashews (≃6.04 Kcal/g), hazelnuts (≃6.71 Kcal/g), macadamias (≃7.51 Kcal/g), pecans (≃7.23 Kcal/g), pine nuts (≃6.01 Kcal/g), pistachios (≃5.95 Kcal/g), and peanuts (≃6.20 Kcal/g) (*Arachis hypogaea*)) are nutrient-dense foods that have been staples of human diets since ancient times and are a defining component of traditional MD. They are now recognized as complex matrices of bioactive macro- and micronutrients, including MUFAs (50–85%) and PUFAs (8–45%), high-quality proteins, fiber, non-sodium minerals, vitamin E, and phytosterols and antioxidant phenolics. These components synergistically promote metabolic and vascular health, along with various other positive health outcomes. Nut consumption has been associated with cholesterol-lowering effects, a reduced incidence of cancer and all-cause mortality, and favorable impacts on cognitive function and depression. Additionally, nuts have yielded modest improvements in glycemic control, blood pressure, endothelial function, and inflammation [153].

The total fat content of nuts, second only to vegetable oils, ranges from 44% in cashews to 76% in macadamias, providing 5.49–7.64 Kcal/g [154,155]. SFA content is low (4–16%), while nearly half of the total fat content is unsaturated fat. MUFAs are predominant in most nuts, whereas PUFAs are more abundant in pine nuts and are nearly equal to the MUFAs found in Brazil nuts. Walnuts are particularly rich in PUFAs, especially α-linolenic acid, the hepatic precursor of EPA and DHA.

Proteins, contributing 8–25% of their energy, are another valuable component of nuts, with significant levels of L-arginine, a key substrate for the synthesis of endothelium-derived nitric oxide (NO), the primary endogenous vasodilator and blood pressure regulator. Nuts also provide dietary fiber, ranging from 0.03 to 0.12 g per 1 g [154,155]. Polyphenols are present in all nuts, with walnuts, pistachios, and pecans having the highest content. Almonds and hazelnuts are rich in vitamin E, while peanuts are notable for their significant vitamin B9 content [154,156].

The fat fraction of nuts contains phytosterols, with pistachios and almonds being particularly rich sources. Evidence suggests that nut-derived phytosterols have cholesterol-lowering effects [157]. Even in lightly salted varieties, nuts have low sodium content. The key represented non-sodium minerals include calcium, magnesium, and potassium. Nut consumption has been linked not only to protection against hypertension, insulin resistance, and CVD [158], but also to the prevention of bone demineralization.

Although nuts are energy-dense foods, evidence from both epidemiological studies and RCTs indicates that regular nut consumption does not predispose individuals to obesity and may even support weight loss [159]. The beneficial effects of nuts on body weight, glycemic control, and CVD suggest a potential positive impact on NAFLD. A few studies have correlated increased nut consumption with a lower incidence of NAFLD [160,161]. Age and gender may influence this relationship, and some studies have reported an inverse association between nut intake and NAFLD [161,162]. Suggested mechanisms underlying the so-far described hepatic protective effects of nut consumption include antioxidant and anti-inflammatory actions.

### 6.6. Extra-Virgin Olive Oil (EVOO)

Regarding seasoning fats, one of the main characteristics of the MD is the regular consumption of EVOO (8.8 kcal/g), a vegetable product obtained from the mesocarp of the fruit of the olive tree (*Olea europaea* L.) through a process of cold pressing. EVOO is widely considered the gold standard of edible oils. It is primarily composed of TGs (97–99%) and is rich in MUFAs (55–83%), mainly oleic acid (OA, C18:1 *n*-9), followed by PUFAs (4–20%, such as linoleic acid) and SFAs (8–14%) [163]. Approximately 1–2% of EVOO’s weight consists of minor components, which include: a non-saponifiable (apolar) fraction comprising squalene, triterpenes, sterols, vitamin E, and pigments, extractable with solvents; a polar fraction, prominently featuring phenolic compounds, to which many of EVOO’s health benefits are attributed [164].

The contents of these components vary, based on factors such as the type of crop, climate, olive maturity at harvest, and the production process used [164]. Among the phenolic compounds, EVOO contains simple phenols (hydroxytyrosol (HT) and tyrosol) and secoiridoids (derivatives of oleuropein and ligstroside) [165]. Additionally, its flavonoid content includes quercetin-3-rutinoside, apigenin-7-rutinoside, apigenin-7-glucoside, luteolin-7-glucoside, and luteolin-5-glucoside.

The unique chemical composition of EVOO has led to its classification as a functional food. Its FAs and bioactive phenolic compounds, both individually and collectively, exert beneficial effects across a wide range of health concerns, including cardiometabolic and neuronal functions, primarily through their anti-inflammatory and antioxidant activities. Based on the available literature, compared to other dietary fats and low-fat diets, EVOO demonstrates superiority in managing clinical biomarkers. It has been shown to lower blood pressure and LDL-C, increase protective HDL cholesterol, improve glycemic control, and aid in weight management [166].

There is substantial evidence that the bioactive compounds of EVOO, such as OA and HT, offer protective effects on the liver in various experimental models, particularly in cellular cultures and animal studies [167,168]. These components significantly reduce hepatic fibrogenesis [169], liver steatosis [168,170], and hepatocyte ballooning. They also prevent lipid peroxidation in rat liver [171] and improve metabolic parameters such as the insulin resistance induced by high-fat diets in animal models [172,173].

A large meta-analysis of 32 prospective observational studies showed that individuals in the upper tertile of olive oil consumption had a 20–40% lower risk of stroke and CVD compared to those in the lower tertile [174]. Other meta-analyses, including both intervention and observational studies, have associated EVOO consumption with a lower risk of diabetes and improvements in metabolic and inflammatory biomarkers [121,122,175]. Recent studies highlight a strong association between high EVOO consumption and a lower prevalence of NAFLD in obese individuals and older adults who are at high cardiovascular risk. These findings are supported by the MICOL study [176] and the PREDIMED study [177].

## 7. Mediterranean Diet and NAFLD: Emerging Mechanisms for Multifaceted Protection

As discussed above, MD as a whole, along with its individual bioactive compounds such as MUFAs, PUFAs, dietary fiber, and polyphenols, can exert protective effects against NAFLD. Multiple mechanisms have been described, and these include the regulation of both intra- and extra-hepatic lipid metabolism, the attenuation of inflammation, the reduction of oxidative stress, and the enhancement of gut health. These metabolic and biochemical effects, either directly or indirectly, can affect liver mitochondrial function, as will be explored later in a dedicated paragraph focusing on how these protective mechanisms converge to support liver mitochondrial function and its critical role in the pathogenesis and potential resolution/prevention of NAFLD.

### 7.1. Monounsaturated Fatty Acids (MUFAs): EVOO and Oleic Acid in NAFLD

Unlike the toxic effects of SFAs, unsaturated fatty acids, particularly MUFAs, exhibit significant benefits for glucose and lipid metabolism in NAFLD. MUFAs, which are central to the MD, prevent hepatic lipid accumulation and improve metabolic health by enhancing plasma lipid profiles, reducing body fat, and modulating adiponectin expression [178]. EVOO, the primary source of MUFAs in the MD, reduces cardiovascular risk, prevents LDL-C oxidation, and improves glycemic control [179,180]. EVOO also increases HDL-mediated macrophage cholesterol efflux and provides antioxidant and anti-inflammatory benefits [181]. OA, the main MUFA in EVOO, has shown promise in NAFLD management by reducing hepatic steatosis, improving insulin sensitivity, and modulating hepatic glucose and lipid metabolism through mechanisms that inhibit ER and oxidative stress and inflammation [182]. Liu et al. [183] further demonstrated OA’s ability to restore autophagic and lysosomal function, reduce apoptosis, and alleviate ER stress in hepatocytes, with in vivo studies showing that olive oil supplementation reverses HFD-induced NAFLD by mitigating ER stress and autophagy dysfunction. These findings emphasize OA’s therapeutic potential in targeting the ER stress–autophagy axis in NAFLD [183].

### 7.2. Omega-3 Polyunsaturated Fatty Acids (PUFAs): Essential Players in Hepatic Lipid Metabolism

Omega-3 PUFAs—including DHA, (EPA, α-linolenic acid, and docosapentaenoic acid (DPA)—are essential nutrients that can be obtained primarily from marine sources like salmon, sardines, and mackerel. Beyond their structural role in cell membranes, omega-3 PUFAs regulate hepatic lipid metabolism, reducing liver fat accumulation, inflammation, and fibrosis while improving insulin sensitivity and counteracting oxidative stress, highlighting their potential as useful agents for managing NAFLD. At the molecular level, they activate the peroxisome proliferator-activated receptor alpha (PPARα) and PGC1α (mainly EPA) to enhance FA oxidation, suppress SREBP-1c and ChREBP (primarily DHA), and inhibit DNL and glycolysis, shifting metabolism from lipid synthesis to oxidation, thereby protecting against hepatic steatosis [184]. Omega-3 PUFAs also mitigate NASH-related inflammation by suppressing NFκB signaling and reducing pro-inflammatory cytokines like TNFα and IL-6 [185,186]. A decline in hepatic C18–22 omega-3 and omega-6 PUFAs and an increased omega-6/omega-3 ratio in NAFLD are linked to inflammation and disease progression [187]. Interestingly, NAFLD may be conceptualized as a condition of omega-3 PUFA deficiency, at least at the hepatic level. This deficiency may result from impaired elongase/desaturase enzyme activity (key to modifying FAs and producing long-chain and unsaturated fatty acids) and fibrotic changes, rather than merely from dietary inadequacy. Notwithstanding this possibility, clinical and preclinical evidence supports omega-3 PUFA supplementation in alleviating NAFLD severity by reducing hepatosteatosis and liver injury. DHA, with its ability to retroconvert to EPA, offers certain advantages, making it a superior option for managing NAFLD (for a comprehensive review, see [188,189]).

Adopting the MD, which is rich in oily fish, EVOO, nuts, and vegetables, can be considered an ideal nutritional model to ensure adequate OA and omega-3 PUFA intake and support liver health, likely promoting a better balance between omega-3 and omega-6 PUFAs while also providing antioxidants and bioactive compounds that help reduce inflammation and oxidative stress, enhancing the benefits for patients with NAFLD [190].

### 7.3. Dietary Fiber: Enhancing Gut Health and Lipid Regulation

Fiber-rich foods in the MD, such as whole cereals, fruits, and vegetables, contribute significantly to NAFLD management by providing water-soluble fibers that modulate lipid metabolism and the gut microbiota. These fibers enhance bile excretion, lowering total serum and LDL-C levels [185]. Epidemiological studies show an inverse relationship between dietary fiber intake and NAFLD prevalence, particularly in individuals with significant fibrosis [186]. Fiber also supports gut microbiota health. Increased fiber intake, especially oligofructose, promotes beneficial microbes like Bifidobacteria, improves gut permeability, and increases short-chain fatty acid (SCFA) production, enhancing metabolic health and reducing dysbiosis [191,192]. SCFAs, including acetate, propionate, and butyrate, maintain gut barrier integrity, alleviate hepatic inflammation, and support hepatic energy balance by regulating appetite and glucose homeostasis. These effects are partly mediated through GPRs, which play a key role in SCFA-regulated processes [193].

### 7.4. Polyphenols and Carotenoids: Targeting Lipid Metabolism, Oxidative Stress, and Inflammation

Extensive preclinical and clinical research has highlighted the pivotal role of polyphenols in targeting key processes such as lipid metabolism, oxidative stress, and inflammation, demonstrating their multifaceted benefits for both healthy and diseased livers.

Polyphenols mitigate hepatic lipid accumulation by modulating key molecular pathways. They downregulate SREBP-1c, inhibiting lipogenesis, and upregulate PPARα, enhancing β-oxidation through AMPK activation [194,195]. Polyphenols also target autophagy via the cAMP/AMPK/SIRT1 pathway, alleviating hepatic steatosis [196]. Additionally, they improve insulin sensitivity, promote hepatic glucose uptake and glycogen synthesis, and reduce the TG accumulation caused by excess glucose or fat intake [197]. EVOO reduces FFA-induced steatosis in HepG2 cells by lowering fat globule size and TG accumulation and mitigates fat accumulation in mice [198]. In HFD-fed rats, EVOO restored PPARα and CPT1 levels, promoting β-oxidation [167]. Moreover, MD adherence improves liver enzymes, notably reducing aspartate aminotransferase (AST) levels without significantly affecting alanine aminotransferase (ALT), as observed by Baratta et al. [199].

Polyphenols also counter oxidative stress, a key factor in NAFLD progression that is driven by ROS and lipid peroxidation products like malondialdehyde and 4-hydroxynonenal [200]. They activate nuclear respiratory factor 2 (Nrf2), inducing antioxidant enzymes to prevent ROS-induced hepatic damage [201]. Inflammation, another driver of NAFLD, is mediated by cytokines like TNF-α, IL-6, and IL-1, which contribute to fibrosis and apoptosis. Polyphenols attenuate these pathways by inactivating NF-κB, reducing cytokine production, and mitigating liver fibrosis and apoptosis [202]. HT reduces pro-inflammatory cytokines like TNF-α, leading to histological improvements in steatosis and inflammation [167].

Among the carotenoids, lycopene has shown promising effects in NAFLD due to its antioxidant, anti-inflammatory, and lipid-lowering properties. It reduces liver steatosis by downregulating the lipogenic genes (acetyl-CoA carboxylase (ACC1), FAS, and SREBP-1c) and enhances antioxidant defense by increasing superoxide dismutase (SOD), catalase (CAT), and glutathione peroxidase (GPx) activity. Lycopene also suppresses inflammation by inhibiting NF-κB and the mitogen-activated protein kinase (MAPK) pathways, leading to decreased TNF-α and IL-1β levels. Clinical and preclinical studies suggest that lycopene supplementation improves liver function, reduces lipid accumulation, and mitigates oxidative stress, making it a potential nutritional strategy for NAFLD management [127].

Notably, polyphenols and carotenoids address multiple pathways, highlighting their potential in NAFLD management within the MD framework. However, their effectiveness remains controversial, due to inconsistent evidence. A recent meta-analysis of RCTs involving 2173 participants found that curcumin reduces the body mass index (BMI), TGs, total cholesterol, liver enzymes, and insulin resistance; catechin reduces BMI, TGs, and insulin resistance; and silymarin lowers liver enzyme levels. In contrast, the efficacy of resveratrol, naringenin, anthocyanin, hesperidin, and catechin requires further RCTs for validation [198,203].

### 7.5. Phytosterols: Modulating Lipid Profiles

Beyond polyphenols, phytosterols, such as stigmasterol, β-sitosterol, and campesterol, which are predominantly found in the cell membranes of plants, particularly in nuts, oilseeds, and legumes, also exhibit significant potential in addressing the multifaceted pathophysiology of NAFLD, with evidence derived from both in vitro and in vivo models. Mechanistically, phytosterols lower total cholesterol and TG levels through multiple pathways. These include competition with dietary cholesterol absorption, the regulation of key enzymes involved in lipid metabolism, the inhibition of hepatic inflammation and oxidative stress, the modulation of liver FA composition, and reductions in cytotoxicity and apoptosis [137,204].

Administration of stigmasterol in mice fed a high-fat and high-cholesterol diet reduced hepatic cholesterol accumulation and enhanced bile acid synthesis via an alternative pathway [205]. Similarly, β-sitosterol has demonstrated protective effects against hepatic steatosis in high-fat or high-fructose diet-fed rats, primarily by modulating lipid metabolism, mitigating inflammation, and alleviating ER stress [206,207]. Furthermore, plant sterol esters of α-linolenic acid have shown efficacy in improving NAFLD by attenuating ER stress-triggered apoptosis and inhibiting pyroptosis. These effects are mediated through the activation of the AMPK and SIRT1 pathways, both in vitro and in vivo, under high-fat dietary conditions [208,209].

Altered phytosterol metabolism, which has been linked to steatosis and inflammation, has been described in obese individuals [210]. Clinical trials have revealed that daily supplementation with phytosterols (1.6–1.8 g) improves lipid profiles, liver enzyme levels, and inflammation by downregulating pro-inflammatory cytokines like TNF-α and enhancing insulin sensitivity [211,212]. Co-supplementation with *n*-3 PUFAs (EPA + DHA) further amplifies these benefits by reducing TG levels, promoting anti-inflammatory lipid mediators, and enhancing membrane phospholipid composition [213]. Metabolomic analyses indicate that phytosterols + omega-3 PUFA supplementation influences key metabolites, such as PUFA-containing phosphatidylcholine and retinyl esters, which may contribute to ameliorated hepatic steatosis [214]. Nonetheless, larger clinical trials are needed to confirm these findings and refine therapeutic strategies.

Mechanistically, the MD’s bioactive compounds offer multifaceted protection against NAFLD. Dietary fiber modulates lipid trafficking and gut health, while phytochemicals and PUFAs regulate lipid metabolism and inflammation. Similarly, MUFAs enhance lipid profiles and reduce oxidative stress. Collectively, these mechanisms address key contributors to NAFLD pathogenesis, including hepatic lipid accumulation, impaired lipophagy, and inflammation—underscoring the therapeutic potential of the MD as a holistic approach to managing liver health. From a clinical perspective, existing data highlight the beneficial effects of the MD in NAFLD patients, showing improvements in BMI, NAFLD scores, hepatic enzymes, and glycemic and lipid profiles [98,101,127,175,176,177,199]. Unfortunately, mechanistic studies at the subcellular level regarding the overall MD remain scarce, leaving critical gaps in our understanding of its comprehensive effects at molecular, biochemical, and metabolic levels, as well as at mitochondrial levels. Moreover, despite strong evidence supporting the MD in NAFLD, several dietary and lifestyle factors may confound its effects, contributing to inconsistencies in human studies. Variability in MD adherence, nutrient interactions (e.g., the omega-6/omega-3 ratio and polyphenol bioavailability), and lifestyle factors such as physical activity, sleep, and alcohol intake can influence metabolic outcomes. Additionally, differences in study design, dietary assessment methods, and population heterogeneity complicate results interpretation. Future research should integrate objective biomarkers, control for confounders, and explore mechanistic links to optimize dietary recommendations for liver health. Furthermore, personalized approaches could enhance the effectiveness of the MD in NAFLD management. Genetic, metabolic, and gut microbiome differences influence individual responses to MD components, including MUFAs, omega-3 PUFAs, and polyphenols. Multi-omics profiling and biomarker-based dietary adjustments may help tailor interventions, optimizing metabolic outcomes. Future clinical trials should integrate personalized nutrition models and use predictive analytics to refine MD-based strategies for NAFLD prevention and treatment.

## 8. Mediterranean Diet and Mitochondria: A New Frontier in NAFLD?

Building upon the evidence of the MD as a cornerstone for promoting liver health, its effect on mitochondrial function is garnering significant scientific attention. Beyond its role in modulating systemic metabolic pathways, the MD may exert profound influence at the mitochondrial level by improving bioenergetics, promoting mitochondrial biogenesis, maintaining dynamic equilibrium through fusion and fission, and enhancing mitophagy. MD’s bioactive components target these mitochondrial processes, offering a mechanistic perspective on their potential to counteract mitochondrial dysfunction in NAFLD.

### 8.1. Mitochondrial Bioenergetics

Polyphenols and other bioactive compounds from the MD play a crucial role in preserving mitochondrial bioenergetics by enhancing ATP production, oxidative phosphorylation, and mitochondrial membrane stability while reducing oxidative stress [215]. Resveratrol, which can be found in peanuts, berries, and grapes, exerts significant effects on mitochondrial metabolism, primarily by modulating sirtuin activity and nicotinamide adenine dinucleotide (NAD)+ availability. A key mechanism involves the modulation of NAD+ levels. Resveratrol activates mitochondrial complex I, enhancing NADH oxidation, which increases the NAD+/NADH ratio. This shift subsequently activates SIRT3, leading to the stimulation of crucial metabolic pathways such as the Krebs cycle, FA β-oxidation, and OXPHOS. These effects have been demonstrated in vitro in isolated enzymes and in HepG2 cells treated with resveratrol (1–5 µmol/L) and demonstrated in vivo in aging mice fed resveratrol (50 mg/kg per day) [216]. Among the carotenoids, lycopene, which is found in red, pink, and orange fruits and vegetables, enhances the expression of cytochrome c and multiple respiratory chain complexes, improving energy efficiency and reducing mitochondrial dysfunction under inflammatory conditions [217]. Supplementation of their diet with polyphenol-rich virgin olive oil in rats resulted in increased hepatic FA oxidation and mitochondrial uncoupling, paralleling reduced H_2_O_2_ release [218]. Combined EPA plus HT supplementation in HFD-mice attenuated liver steatosis-associated alterations, among which were decreased FA oxidation, mitochondrial respiratory capacity, and ATP content [219]. Badolati et al. [220] tested the protective effect exerted on the liver by the antioxidant Taurisolo, a nutraceutical formulation produced from grape pomace and enriched with resveratrol and other polyphenols. Both in vitro (in cultured hepatic HuH7 cells) and in vivo (in high-fat diet-fed mice), Taurisolo reduced the markers of NAFLD, increasing mitochondrial activity, promoting respiration and ATP production, and fostering FA β-oxidation [220]. Recently, Beatriz Sánchez-Calvo and coworkers used HFD-fed mice to test the effect of dietary EVOO supplementation in the presence of NO_2_^−^, intended to stimulate NO_2_-fatty acid formation during digestion, mimicking the spontaneous intake of EVOO and vegetables rich in NO_2_^−^ and nitrate, as seen in humans following the MD. Experimental conditions stimulating NO_2_-fatty acid formation significantly improved mitochondrial functions in both state 3 and RCR, and increased Complex II (succinate dehydrogenase) and Complex V (ATP synthase) activities [221].

These findings underscore the MD’s potential to support energy metabolism and maintain bioenergetic stability during metabolic stress, strengthening the importance of combined interventions and considering raw food effects.

### 8.2. Mitochondrial Biogenesis

MD-derived compounds also promote liver mitochondrial biogenesis (for a recent review, see [222,223]). EVOO’s HT enhances mitochondrial abundance by inducing the expression of biogenesis regulators, including PGC-1α, mitochondria transcription factor A (TFAM), and nuclear respiratory factor 1 (NRF1), achieved through AMPK pathway activation [224,225]. Resveratrol promotes biogenesis via the NAD-dependent deacetylase sirtuin-1 (SIRT1)-mediated activation of PGC-1α and improves mitochondrial function, particularly in a high-calorie diet or dysmetabolism (i.e., intrauterine growth retardation) models [226,227,228].

In vitro and in vivo studies also revealed stimulatory effects on mitochondrial biogenesis for quercetin, one of the most abundant dietary flavonoids found in fruits (mainly citrus), green leafy vegetables, and many seeds. In HepG2 cells, quercetin activates PGC-1α [229] and promotes the overexpression of cytochrome oxidase subunit IV (COXIV) [230], concomitantly increasing other mitochondrial proteins involved in the respiratory chain, Krebs cycle, and FA oxidation pathways, resulting in an overall enhancement of mitochondrial capacity [230,231]. In animal models, quercetin doses of 50 mg/kg and 100 mg/kg have demonstrated significant effects on mitochondrial biogenesis, with no observed toxicity, even at doses as high as 3000 mg/kg [231].

In ob/ob mice, curcumin (1% or 3%, *w*/*w*), a yellow polyphenolic pigment found in saffron, turmeric, and ginger that is increasingly used in the modern Mediterranean kitchen, enhanced the expression of Nrf1 and Tfam, with a concomitant increase in mitochondrial respiratory chain complex I activity and ATP levels, preventing liver steatosis [232]. Additionally, in diabetic db/db mice, oral curcumin administration at a dose of 60 mg/kg/day for 4 weeks ameliorated mitochondrial dysfunction by decreasing lipid peroxidation and nitric oxide synthesis, while enhancing mitochondrial ATPase activity in liver-isolated mitochondria [233]. More recently, curcumin administration to postnatal overfed rats resulted in preserved liver mitochondrial biogenesis and antioxidant response and upregulated mRNA and the protein expression of SIRT3. Curcumin also decreased the levels of cellular lipids and mitochondrial reactive oxygen species in vitro in L02 cells exposed to FFAs, where it succeeded in increasing the mt-DNA copy number and SOD activity [234].

### 8.3. Mitochondrial Dynamics

MD components regulate mitochondrial dynamics, including fission, fusion, and mitophagy, and this may contribute to maintaining mitochondrial quality and function. HT has been shown to reduce fission by downregulating DRP1 and PPAR while promoting mitochondrial health [224]. Ferulic acid, which is found in fruits, vegetables, and plants such as apples, oranges, coffee, artichokes, wheat, and oats, modulates mitochondrial dynamics by stimulating the expression of MFN1, MFN2, and fission protein 1 (FIS1), ensuring a balance between the fusion and fission processes [235]. Additionally, the SCFA butyrate improves mitochondrial fusion and suppresses fission by enhancing mitochondrial fusion protein mitofusin-1 (MFN1), MFN2, and optic atrophy 1 (OPA1) expression while reducing DRP1 levels [236]. Together, these effects maintain mitochondrial integrity and prevent dysfunction in metabolic stress conditions.

### 8.4. Mitophagy

While polyphenols from the MD have been shown to enhance general autophagy, evidence now also suggests specific roles in mitophagy.

Quercetin activates the transcription factor Forkhead box protein O3 (FOXO3a) via AMPK and extracellular signal-regulated kinase 2 (ERK2) signaling, restoring mitophagy in ethanol-induced liver injury and reducing mitochondrial oxidative stress [237]. Both in vitro and in vivo, quercetin was shown to dose-dependently alleviate NAFLD effectively through AMPK-PINK1 Parkin-mediated mitophagy [238,239]. Cyanidin-3-*O*-glucoside, one of the most widely distributed anthocyanins in red and blue fruits, may also alleviate NAFLD by activating PINK1-mediated mitophagy. Data have been obtained in human, rodent, and in vitro models. Cyanidin-3-*O*-glucoside has been shown to (i) inhibit hepatic lipid accumulation and improve insulin sensitivity in mice; (ii) suppress lipid accumulation and upregulate the expression of autophagosome formation genes and of PINK1, Parkin, and translocase of the outer membrane 20 (TOM20) in palmitic acid-induced alpha mouse liver 12 cells; (iii) decrease TGs and increase the expressions of PINK1, Parkin, and TOM20 in hepatocytes from NAFLD patients [240].

EVOO polyphenols, such as HT, alleviate liver fat accumulation, oxidative stress, and mitochondrial dysfunction by activating mitophagy through the AMPK/PINK1 pathway in fish [241].

The Mediterranean diet’s bioactive compounds, including polyphenols, omega-3 PUFAs, and SCFAs, exhibit multifaceted benefits targeting mitochondria. The mechanisms involved collectively contribute to maintaining mitochondrial health, which is central to mitigating oxidative stress, inflammation, and metabolic dysfunction in NAFLD and related conditions. Future research should further elucidate the precise molecular pathways through which these compounds exert their effects, particularly in human clinical models, to optimize MD-based interventions for NAFLD. For a schematic summary of the mechanisms discussed above, see Table 1 and Table 2.

## 9. Fructose and NAFLD: Friend or Foe?

Given the central role of mitochondria in NAFLD pathogenesis, it is crucial to examine how fructose, a key dietary component of the MD, influences mitochondrial function and liver metabolic health. Indeed, this monosaccharide, which is naturally present in fruits, may hold a controversial position within the MD. While the excessive intake of added fructose—commonly found in processed foods and beverages—has been strongly linked to DNL, hepatic steatosis, and NAFLD progression [242], fructose derived from whole fruits may exhibit a different metabolic profile. In the context of the MD, fruits provide not only fructose but also a matrix of dietary fiber, polyphenols, vitamins, and minerals, which collectively mitigate fructose’s potentially deleterious effects by slowing its absorption, enhancing satiety, and exerting antioxidant and anti-inflammatory actions. Nevertheless, the dose and source of fructose remain critical factors, and further research is warranted to fully elucidate its role in the MD and its impact on liver health.

Recent clinical and observational studies present conflicting evidence on the effect of fruit consumption on NAFLD. A randomized controlled trial investigating the effect of a fruit-rich diet (FRD) in NAFLD patients found that consuming more than four servings of fruit daily led to the worsening of hepatic steatosis, insulin resistance, and dyslipidemia [243]. The study observed increased BMI, higher liver enzyme levels, and worsened glycemic control in the high-fruit group, suggesting that excessive fructose intake, even from whole fruit, could exacerbate metabolic dysfunction in NAFLD patients.

In contrast, a cross-sectional study in Chinese patients with T2D found that a high vegetable intake was significantly associated with a reduced risk of NAFLD, whereas fruit intake did not show a strong inverse association [244]. Similarly, a meta-analysis of observational studies indicated that both higher vegetable and fruit consumption were inversely correlated with NAFLD risk, but the effect size varied across populations [245]. These findings imply that while higher overall fruit and vegetable intake may be beneficial, specific types of fruits or excessive fructose consumption could counteract these benefits.

Further evidence suggests that certain fruits rich in bioactive phytoconstituents may exert protective effects against NAFLD. A randomized clinical trial found that daily orange consumption reduced hepatic steatosis prevalence in patients with metabolic dysfunction-associated steatotic liver disease, independent of body weight changes [246]. This suggests that flavonoid-rich citrus fruits may offer hepatoprotective benefits, possibly through their antioxidant and anti-inflammatory properties.

From a mechanistic perspective, fructose metabolism differs significantly from glucose metabolism. Unlike glucose, which is tightly regulated by insulin, fructose is primarily metabolized in the liver, where it bypasses key regulatory steps, leading to increased TG synthesis and potential hepatic fat accumulation. Studies in animal models have shown that high-fructose diets upregulate lipogenic genes such as SREBP-1c and ACC, promote insulin resistance, and induce hepatic inflammation [247]. However, the presence of the fiber and polyphenols found in whole fruits may counteract these effects by modulating the gut microbiota, enhancing insulin sensitivity, and reducing oxidative stress [248].

Emerging evidence suggests that high-fructose diets induce mitochondrial dysfunction. Proteomic analyses have revealed that fructose consumption alters the abundance of mitochondrial proteins involved in β-oxidation and OXPHOS, contributing to mitochondrial inefficiency, lipid accumulation, and oxidative stress, thereby accelerating NAFLD pathogenesis [249]. Additionally, fructose has been shown to induce oxidative damage to mt-DNA while also impairing its repair mechanisms, leading to a reduced mt-DNA copy number and defective mitochondrial biogenesis. This mitochondrial dysfunction exacerbates liver injury, promoting inflammation and fibrosis [250,251]. Notably, new evidence suggests that, in the context of high fat levels, fructose may influence hepatic mitochondrial dynamics by impairing the fusion–fission balance, leading to fragmented and dysfunctional mitochondria, a hallmark of metabolic disease progression [252]. The fundamental role of epigenetic factors such as microRNAs (miRNAs) is also emerging. A recent study reported that fructose overconsumption in rats affected liver miR-125b-5p expression levels and the miR-34a-5p/SIRT-1: AMPK pathway, resulting in a decrease in fat oxidation and an increase in fat synthesis and DNL [253].

While excessive fructose intake is harmful, certain dietary components may counteract its negative effects. Bioactive compounds found in fruits, such as polyphenols, have demonstrated hepatoprotective properties. For instance, pomegranate-derived polyphenols have been shown to restore mitochondrial homeostasis and strengthen endogenous antioxidant defenses in fructose-fed animal models [249]. These findings suggest that while fructose itself can contribute to NAFLD pathology, its effects may be mitigated by co-consumption with bioactive-rich foods, as found in a balanced MD.

The role of fructose in NAFLD remains complex and context-dependent. While excessive fructose intake from processed foods and beverages is clearly detrimental, the moderate consumption of whole fruits within a balanced MD may be beneficial or neutral, depending on the type of fruit, overall dietary composition, and individual metabolic health status. Future research should focus on identifying the specific fruit types and the bioactive components that contribute to either beneficial or harmful outcomes in NAFLD patients. Furthermore, investigating how fructose interacts with mitochondrial function in the context of a polyphenol-rich diet could offer deeper insights into dietary strategies for NAFLD management. Until then, dietary recommendations should emphasize moderation and variety, favoring fiber-rich, polyphenol-dense fruits while avoiding the excessive intake of high-fructose fruits in metabolically compromised individuals.

## 10. Conclusions

To sum up, promising evidence from preclinical and clinical studies supports the MD and its bioactive compounds as a non-pharmacological approach to preventing and managing NAFLD. Adherence to the MD and the use of individual or co-administered bioactive compounds have been shown to beneficially impact key variables such as liver fat content, hepatic enzymes, anthropometric measures, and metabolic markers. Nonetheless, the precise molecular mechanisms, particularly the role of mitochondria in MD-mediated improvements in NAFLD, remain incompletely elucidated.

Possible pathways that have been investigated in both in vitro and in vivo models include mitochondrial fuel substrate control, OXPHOS efficiency, mitochondrial dynamics, and biogenesis. Bioactive compounds found in the MD, such as polyphenols, omega-3 PUFA, and flavonoids, have demonstrated the potential to enhance mitochondrial function by reducing oxidative stress, improving mitophagy, and modulating key regulators of energy metabolism. However, the interplay between the whole diet, mitochondrial function, and NAFLD progression is complex and is influenced by factors such as genetic predisposition, gut microbiota composition, and metabolic status.

To address these gaps, future research should adopt a multidisciplinary approach combining advanced experimental and clinical methodologies. In preclinical studies, future approaches should integrate multi-omics approaches with high-resolution mitochondrial imaging and functional assays to precisely map the mitochondrial adaptations induced by the MD and its bioactive compounds. The use of human organoid models and patient-derived hepatocytes could offer physiologically relevant insights into dietary–mitochondrial interactions.

In clinical research, longitudinal dietary intervention trials with well-characterized MD adherence scores should be combined with in vivo assessments of mitochondrial function. Non-invasive techniques and metabolomic profiling could help track mitochondrial energetics and lipid metabolism in NAFLD patients. Further research is warranted to clarify the optimal dietary composition and bioactive combinations that exert the most potent hepatoprotective effects. Comparative clinical trials investigating whole-diet MD adherence versus specific nutrient supplementation will be crucial to determine whether synergistic effects exist. Additionally, the development of precision nutrition strategies incorporating mitochondrial biomarkers (e.g., circulating mtDNA) and metabolic phenotyping may help tailor MD-based recommendations for individuals with NAFLD.

Overall, while MD offers a promising, mitochondria-centered nutritional strategy for NAFLD, a deeper mechanistic understanding is essential for its clinical translation. Future research should build upon existing findings by integrating high-resolution experimental techniques with well-designed human trials. This approach will help refine therapeutic guidelines and optimize metabolic health outcomes.

## Figures and Tables

**Table 1:** Main hepato-protective and mitochondrial effects of MD bioactive compounds.

	BIOACTIVES				
	MUFAs	PUFAs	FIBERS	PHYTOSTEROLS	CAROTENOIDS
HEPATIC EFFECTS	prevent hepatic lipid accumulation [178] increase HDL-mediated macrophage cholesterol efflux [181] modulate adiponectin expression [178] elicit antioxidant and anti-inflammatory benefits [181] **OA** modulates hepatic glucose and lipid metabolism [182] inhibits ER and oxidative stress, and inflammation [182] restores autophagic and lysosomal function [183] reduces apoptosis [183]	prevent hepatic lipid accumulation [184] regulate hepatic lipid metabolism [184] act on PPARα and PGC1α [184] suppress SREBP-1c and ChREBP inhibit DNL and glycolysis [184] reduce oxidative stress [184] reduce fibrosis and inflammation [188,189] **OMEGA 3:** suppress NFKβ signaling [185,186] reduce cytokines level as TFNα and IL-6 [185,186] **OMEGA 6:** activate AMPK and SIRT1 [208,209] attenuate ER stress-triggered apoptosis [208,209]	enhance hepatic metabolic health [185] increase bile excretion [185] **SCFAs** support hepatic energy balance [193] alleviate hepatic inflammation [193]	improve lipid profiles [211,212] reduce inflammation [211,212] down-regulate TNF-α [211,212] enhance insulin sensitivity [211,212] **STIGMASTEROL** reduces hepatic cholesterol accumulation [205] enhances bile acid synthesis [205] **β-sisterol** alleviates hepatic steatosis [206,207] modulates lipid metabolism [206,207] mitigates inflammation [206,207] activates AMPK and SIRT1 [208,209] attenuates ER stress-triggered apoptosis [208,209]	**LYCOPENE** improves liver function [127] has anti-inflammatory and lipid-lowering properties [127] downregulates ACC1, FAS, SREBP-1c [127] inhibits NF-Kβ and MAPK [127] elicits antioxidant effects [127] enhances CAT, GPx protein expression levels [127]
MITOCHONDRIAL EFFECTS		enhance β-oxidation [184] **EPA+HT:** increase mitochondrial respiratory capacity [219] increase ATP content [219]	**SCFAs** increase expression of MFN1, MFN2 and OPA1 [236] reduce expression of DRP1 [236]		**LYCOPENE** improves energy efficiency [217] reduces mitochondrial disfunction [217] enhances SOD protein expression [127] increase expression of cytochrome C [217]

**Table 2:** Main hepato-protective and mitochondrial effects of key MD polyphenols.

	POLYPHENOLS
**HEPATIC EFFECTS**	alleviate hepatic steatosis [194,195] inhibit lipogenesis [194,195] downregulate SREBP-1c [194,195] upregulate PPARα [194,195] target cAMP/AMPK/SRT1 pathway [196] improve inflammation [167] reduce levels of IL-6 and TNFα [167] promote hepatic glucose uptake and glycogen synthesis [197] reduce oxidative stress [200] activate NRF2-induced antioxidant enzymes [201] **EVOO POLYPHENOLS** alleviate liver fat accumulation [241] inhibit TG accumulation [198] reduce oxidative stress [241] **QUERCETIN** activates expression of FOXO3a via AMPK and ERK2 signaling [237] **CYANIDIN-3-O-glucoside** inhibits lipid accumulation [240] improve insulin sensitivity [240] **TAURISOLO** reduced markers of NAFLD [220] **CURCUMIN** decreases lipid peroxidation [233] reduces levels of cellular lipids [234]
**MITOCHONDRIAL** **EFFECTS**	enhance β-oxidation through AMPK activation [194,195] enhance ATP production [216] **EVOO POLYPHENOLS** improve hepatic β-oxidation [167,218] restore PPARα and CPT1 levels [198] reduce H_2_O_2_ release [218] increase mitochondrial uncoupling [218] enhance expression of PGC1α, TFAM, NRF1, AMPK [224,225] alleviate mitochondrial dysfunction via the activation of AMPK/PINK pathway [241] down-regulate DRP1 and PPAR expression [224] **RESVASTROL** improves mitochondrial function [226,227,228] stimulates Krebs cycle, β-oxidation and OXPHOS activity [216] promotes biogenesis via SIRT1-mediated activation of PGC1α [226,227,228] regulates NAD availability [216] activates mitochondrial respiratory complex I [216] enhances NADH oxidation [216] **QUERCETIN** stimulates Krebs cycle, β-oxidation and OXPHOS activity [230,231] activates PGC1α [230] promotes expression of mitochondrial respiratory complex IV [230] activates AMPK-PINK1-PARKIN pathway [238,239] **CYANIDIN-3-O-glucoside** increases expression of PARKIN, TOM20, PINK1 [240] **FERULIC ACID** stimulates expression of MFN1, MFN2 and FIS1 [235] **TAURISOLO** fosters β-oxidation [220] increases mitochondrial activity and ATP production [220] **CURCUMIN** preserves liver mitochondrial biogenesis [234] enhances expression of NRF1 and TFAM [232] increases activity of mitochondrial respiratory complex I [232] enhances mitochondrial ATPase activity [233] increase mt-DNA copy number [234] ameliorate antioxidant response via SITR3 [234] reduces mitochondrial ROS [234] induces SOD activity [234]

## Abbreviations

The following abbreviations are used in this manuscript:

ACC1	acetyl-CoA carboxylase
ALT	alanine aminotransferase
AMPK	5′ AMP-activated protein kinase
apoB100	apolipoprotein B100
AST	aspartate aminotransferase
ATG5	autophagy protein 5
BMI	body mass index
BNIP3	BCL2/adenovirus E1B 19 kDa protein-interacting protein 3
CAT	catalase
CD36	cluster of differentiation 36
CHREBP1	carbohydrate response element binding protein 1
COXIV	cytochrome oxidase subunit IV
CRC	colorectal cancer
CVD	cardiovascular disease
CYP2E1	cytochrome P450 2E1
DAG	diacylglycerol
DASH	dietary approaches to stop hypertension
DHA	docosahexaenoic acid
DNL	de novo lipogenesis
DPA	docosapentaenoic acid
DRP1	dynamin-related protein 1
EASL	European Association for the Study of the Liver
ECM	extracellular matrix
Eno	endothelial nitric oxide synthase
EPA	eicosapentaenoic acid
ER	endoplasmic reticulum
ERK2	extracellular signal-regulated kinase 2
FAS	fatty acid synthase
FAs	fatty acids
FATP2	fatty acid transport protein 2
FATP5	fatty acid transport protein 5
FFAs	free fatty acids
FIS1	fission protein 1
FMT	fecal microbiota transplantation
FOXO3a	transcription factor forkhead box protein O3
FRD	fruit-rich diet
FXR	farnesoid X receptor
GCKR	glucokinase (hexokinase 4) regulator
GLP-1	glucagon-like peptide 1
GPX	glutathione peroxidase
H_2_O_2_	hydrogen peroxide
HCC	hepatocellular carcinoma
HDAC6	histone deacetylase 6
HDL	low high-density lipoprotein
HSCs	hepatic stellate cells
HT	hydroxytyrosol
IL-1β	interleukin 1β
IL-6	interleukin 6
LC3	microtubule-associated protein 1A/1B-light chain 3
LDL-C	Low-density lipoprotein cholesterol
LPS	lipopolysaccharides
LXRα	liver X receptor alpha
MAPK	mitogen-activated protein kinase
MD	Mediterranean diet
MFN1	mitochondrial fusion protein mitofusin-1
MFN2	mitochondrial fusion protein mitofusin-2
MQC	quality control mechanisms
Mt-DNA	mitochondrial DNA
MTTP	microsomal triglyceride transfer protein
MUFAs	monounsaturated fatty acids
NAD	nicotinamide adenine dinucleotide
NAFLD	non-alcoholic fatty liver disease
NASH	Non-alcoholic steatohepatitis
NBR1	neighbor of BRCA1 gene 1
NFKβ	nuclear factor K chain transporter
NHANES	National Health and Examination Survey
NLRP3	NLR family pyrin domain containing 3
NO	endothelium-derived nitric oxide
NRF1	nuclear respiratory factor 1
NRF2	nuclear respiratory factor 2
OA	oleic acid
OMM	outer mitochondrial membrane
OPA1	optic atrophy 1
OXPHOS	oxidative phosphorylation
p62	ubiquitin-binding protein p62
PDGF	platelet-derived growth factor
PGC1α	peroxisome proliferator-activated receptor gamma coactivator 1-alpha
PINK1	PTEN-induced kinase 1
PKCε	protein kinase C epsilon
PNPLA3	patatin-like phospholipase domain-containing protein-3
PPAR-α	peroxisome proliferator-activated receptor alpha
PPAR-γ	proliferator-activated receptor gamma
PS	phytosterols
PUFAs	polyunsaturated fatty acids
ROS	reactive oxygen species
SCD1	stearoyl-CoA desaturase 1
SCFA	short-chain fatty acid
SFAs	saturated fatty acids
SGLT2i	sodium-glucose transporter protein 2 inhibitors
SIRT1	NAD-dependent deacetylase sirtuin-1
SIRT3	NAD-dependent deacetylase sirtuin-3
SOD	superoxide dismutase
SREBP1c	transcription factor sterol regulatory element binding protein-1c
T2D	Type 2 diabetes
Tfam	mitochondria transcription factor A
TG	triglyceride
TGFβ1	transforming growth factor β1
TGR5	Takeda G protein-coupled receptor 5
TGs	triglycerides
TLR4	toll-like receptor 4
TM6SF2	transmembrane 6 superfamily 2 human gene
TNFα	tumor necrosis factor α
TOM20	translocase of the outer membrane 20
ULK1	unc-5-like autophagy-activating kinase 1
VLDL	very-low-density lipoprotein levels

## References

[1] Chalasani N., Younossi Z., Lavine J.E., Charlton M., Cusi K., Rinella M., Harrison S.A., Brunt E.M., Sanyal A.J. (2018). The diagnosis and management of nonalcoholic fatty liver disease: Practice guidance from the American Association for the study of Liver diseases. Hepatology.

[2] Zelber-Sagi S., Ratziu V., Oren R. (2011). Nutrition and physical activity in NAFLD: An overview of the epidemiological evidence. World J. Gastroenterol..

[3] Bakhshimoghaddam F., Baez D., Dolatkhah N., Sheikh M., Poustchi H., Hekmatdoost A., Dawsey S., Kamangar F., Abnet C., Malekzadeh R. (2024). Which dietary patterns fend off nonalcoholic fatty liver disease? A systematic review of observational and interventional studies. BMC Nutr..

[4] Torres-Peña J.D., Arenas-de Larriva A.P., Alcala-Diaz J.F., Lopez-Miranda J., Delgado-Lista J. (2023). Different Dietary Approaches, Non-Alcoholic Fatty Liver Disease and Cardiovascular Disease: A Literature Review. Nutrients.

[5] European Association for the Study of the Liver (EASL), European Association for the Study of Diabetes (EASD), European Association for the Study of Obesity (EASO) (2016). EASL-EASD-EASO Clinical Practice Guidelines for the management of non-alcoholic fatty liver disease. J. Hepatol..

[6] Younossi Z.M., Corey K.E., Lim J.K. (2021). AGA Clinical Practice Update on Lifestyle Modification Using Diet and Exercise to Achieve Weight Loss in the Management of Nonalcoholic Fatty Liver Disease: Expert Review. Gastroenterology.

[7] Plauth M., Bernal W., Dasarathy S., Merli M., Plank L.D., Schütz T., Bischoff S.C. (2019). ESPEN guideline on clinical nutrition in liver disease. Clin. Nutr..

[8] Berná G., Romero-Gomez M. (2020). The Role of Nutrition in Non-Alcoholic Fatty Liver Disease: Pathophysiology and Management. Liver Int..

[9] Rudrapal M., Khairnar S.J., Khan J., Dukhyil A.B., Ansari M.A., Alomary M.N., Alshabrmi F.M., Palai S., Deb P.K., Devi R. (2022). Dietary polyphenols and their role in oxidative stress-induced human diseases: Insights into protective effects, antioxidant potentials and Mechanism(s) of action. Front. Pharmacol..

[10] Grattagliano I., Di Ciaula A., Baj J., Molina-Molina E., Shanmugam H., Garruti G., Wang D.Q., Portincasa P. (2021). Protocols for Mitochondria as the Target of Pharmacological Therapy in the Context of Nonalcoholic Fatty Liver Disease (NAFLD). Methods Mol. Biol..

[11] Raza S., Rajak S., Upadhyay A., Tewari A., Anthony Sinha R. (2021). Current treatment paradigms and emerging therapies for NAFLD/NASH. Front. Biosci..

[12] Pafili K., Roden M. (2021). Nonalcoholic fatty liver disease (NAFLD) from pathogenesis to treatment concepts in humans. Mol. Metab..

[13] Cotter T.G., Rinella M. (2020). Nonalcoholic fatty liver disease 2020: The State of the disease. Gastroenterology.

[14] Pafili K., Maltezos E., Papanas N. (2018). Ipragliflozin and sodium glucose transporter 2 inhibitors to reduce liver fat: Will the prize we sought be won?. Expert Opin. Pharmacother..

[15] Kechagias S., Ekstedt M., Simonsson C., Nasr P. (2022). Non-invasive diagnosis and staging of non-alcoholic fatty liver disease. Hormones.

[16] Sookoian S., Pirola C.J. (2011). Meta-analysis of the influence of I148M variant of patatin-like phospholipase domain containing 3 gene (PNPLA3) on the susceptibility and histological severity of nonalcoholic fatty liver disease. Hepatology.

[17] Dongiovanni P., Petta S., Maglio C., Fracanzani A.L., Pipitone R., Mozzi E., Motta B.M., Kaminska D., Rametta R., Grimaudo S. (2015). Transmembrane 6 superfamily member 2 gene variant disentangles nonalcoholic steatohepatitis from cardiovascular disease. Hepatology.

[18] Petta S., Miele L., Bugianesi E., Cammà C., Rosso C., Boccia S., Cabibi D., Di Marco V., Grimaudo S., Grieco A. (2014). Glucokinase regulatory protein gene polymorphism affects liver fibrosis in non-alcoholic fatty liver disease. PLoS ONE.

[19] Miquilena-Colina M.E., Lima-Cabello E., Sánchez-Campos S., García-Mediavilla M.V., Fernández-Bermejo M., Lozano-Rodríguez T., Vargas-Castrillón J., Buqué X., Ochoa B., Aspichueta P. (2011). Hepatic fatty acid translocase CD36 upregulation is associated with insulin resistance, hyperinsulinaemia and increased steatosis in non-alcoholic steatohepatitis and chronic hepatitis C. Gut.

[20] Enooku K., Tsutsumi T., Kondo M., Fujiwara N., Sasako T., Shibahara J., Kado A., Okushin K., Fujinaga H., Nakagomi R. (2020). Hepatic FATP5 expression is associated with histological progression and loss of hepatic fat in NAFLD patients. J. Gastroenterol..

[21] Hernández E.Á., Kahl S., Seelig A., Begovatz P., Irmler M., Kupriyanova Y., Nowotny B., Nowotny P., Herder C., Barosa C. (2017). Acute dietary fat intake initiates alterations in energy metabolism and insulin resistance. J. Clin. Investig..

[22] Bortolotti M., Kreis R., Debard C., Cariou B., Faeh D., Chetiveaux M., Ith M., Vermathen P., Stefanoni N., Lê K.A. (2009). High protein intake reduces intrahepatocellular lipid deposition in humans. Am. J. Clin. Nutr..

[23] Ngo Sock E.T., Le K.A., Ith M., Kreis R., Boesch C., Tappy L. (2010). Effects of a short-term overfeeding with fructose or glucose in healthy young males. Br. J. Nutr..

[24] Lê K.A., Ith M., Kreis R., Faeh D., Bortolotti M., Tran C., Boesch C., Tappy L. (2009). Fructose overconsumption causes dyslipidemia and ectopic lipid deposition in healthy subjects with and without a family history of type 2 diabetes. Am. J. Clin. Nutr..

[25] Carpino G., Del Ben M., Pastori D., Carnevale R., Baratta F., Overi D., Francis H., Cardinale V., Onori P., Safarikia S. (2020). Increased Liver Localization of Lipopolysaccharides in Human and Experimental NAFLD. Hepatology.

[26] Zhang X., Ji X., Wang Q., Li J.Z. (2018). New insight into inter-organ crosstalk contributing to the pathogenesis of non-alcoholic fatty liver disease (NAFLD). Protein Cell.

[27] Swann J.R., Want E.J., Geier F.M., Spagou K., Wilson I.D., Sidaway J.E., Nicholson J.K., Holmes E. (2011). Systemic gut microbial modulation of bile acid metabolism in host tissue compartments. Proc. Natl. Acad. Sci. USA.

[28] Polyzos S.A., Kountouras J., Mantzoros C.S. (2016). Adipokines in nonalcoholic fatty liver disease. Metabolism.

[29] Roden M., Shulman G.I. (2019). The integrative biology of type 2 diabetes. Nature.

[30] Lambert J.E., Ramos-Roman M.A., Browning J.D., Parks E.J. (2014). Increased de novo lipogenesis is a distinct characteristic of individuals with nonalcoholic fatty liver disease. Gastroenterology.

[31] Marques-Lopes I., Ansorena D., Astiasaran I., Forga L., Martinez J.A. (2001). Postprandial de novo lipogenesis and metabolic changes induced by a high-carbohydrate, low-fat meal in lean and overweight men. Am. J. Clin. Nutr..

[32] Benhamed F., Denechaud P.D., Lemoine M., Robichon C., Moldes M., Bertrand-Michel J., Ratziu V., Serfaty L., Housset C., Capeau J. (2012). The lipogenic transcription factor ChREBP dissociates hepatic steatosis from insulin resistance in mice and humans. J. Clin. Investig..

[33] Higuchi N., Kato M., Shundo Y., Tajiri H., Tanaka M., Yamashita N., Kohjima M., Kotoh K., Nakamuta M., Takayanagi R. (2008). Liver X receptor in cooperation with SREBP-1c is a major lipid synthesis regulator in nonalcoholic fatty liver disease. Hepatol. Res..

[34] Magkos F., Su X., Bradley D., Fabbrini E., Conte C., Eagon J.C., Varela J.E., Brunt E.M., Patterson B.W., Klein S. (2012). Intrahepatic diacylglycerol content is associated with hepatic insulin resistance in obese subjects. Gastroenterology.

[35] Luukkonen P.K., Zhou Y., Sädevirta S., Leivonen M., Arola J., Orešič M., Hyötyläinen T., Yki-Järvinen H. (2016). Hepatic ceramides dissociate steatosis and insulin resistance in patients with non-alcoholic fatty liver disease. J. Hepatol..

[36] Bhatti J.S., Bhatti G.K., Reddy P.H. (2017). Mitochondrial dysfunction and oxidative stress in metabolic disorders—A step towards mitochondria based therapeutic strategies. Biochim. Biophys. Acta Mol. Basis Dis..

[37] Koliaki C., Szendroedi J., Kaul K., Jelenik T., Nowotny P., Jankowiak F., Herder C., Carstensen M., Krausch M., Knoefel W.T. (2015). Adaptation of hepatic mitochondrial function in humans with non-alcoholic fatty liver is lost in steatohepatitis. Cell Metab..

[38] Shami G.J., Samarska I.V., Koek G.H., Li A., Palma E., Chokshi S., Wisse E., Braet F. (2023). Giant mitochondria in human liver disease. Liver Int..

[39] Cortez-Pinto H., Chatham J., Chacko V.P., Arnold C., Rashid A., Diehl A.M. (1999). Alterations in liver ATP homeostasis in human nonalcoholic steatohepatitis: A pilot study. JAMA Netw..

[40] Traussnigg S., Kienbacher C., Gajdošík M., Valkovič L., Halilbasic E., Stift J., Rechling C., Hofer H., Steindl-Munda P., Ferenci P. (2017). Ultra-high-field magnetic resonance spectroscopy in non-alcoholic fatty liver disease: Novel mechanistic and diagnostic insights of energy metabolism in non-alcoholic steatohepatitis and advanced fibrosis. Liver Int..

[41] Kazankov K., Jørgensen S.M.D., Thomsen K.L., Møller H.J., Vilstrup H., George J., Schuppan D., Grønbæk H. (2019). The role of macrophages in nonalcoholic fatty liver disease and nonalcoholic steatohepatitis. Nat. Rev. Gastroenterol. Hepatol..

[42] Were A., McGeough M.D., Peña C.A., Schlattjan M., Li H., Inzaugarat M.E., Messer K., Canbay A., Hoffman H.M., Feldstein A.E. (2014). NLRP3 inflammasome activation is required for fibrosis development in NAFLD. J. Mol. Med..

[43] Wobser H., Dorn C., Weiss T.S., Amann T., Bollheimer C., Büttner R., Schölmerich J., Hellerbrand C. (2009). Lipid accumulation in hepatocytes induces fibrogenic activation of hepatic stellate cells. Cell Res..

[44] Zehra M., Curry J.C., Pillai S.S., Lakhani H.V., Edwards C.E., Sodhi K. (2020). Elucidating potential profibrotic mechanisms of emerging biomarkers for early prognosis of hepatic fibrosis. Int. J. Mol. Sci..

[45] Xu L., Hui A.Y., Albanis E., Arthur M.J., O’Byrne S.M., Blaner W.S., Mukherjee P., Friedman S.L., Eng F.J. (2005). Human hepatic stellate cell lines, LX-1 and LX-2: New tools for analysis of hepatic fibrosis. Gut.

[46] Haghgoo S.M., Sharafi H., Alavian S.M. (2019). Serum cytokines, adipokines and ferritin for non-invasive assessment of liver fibrosis in chronic liver disease: A systematic review. Clin. Chem. Lab. Med..

[47] Singh R., Kaushik S., Wang Y., Xiang Y., Novak I., Komatsu M., Tanaka K., Cuervo A.M., Czaja M. (2009). Autophagy regulates lipid metabolism. Nature.

[48] Adiels M., Taskinen M.R., Packard C., Caslake M.J., Soro-Paavonen A., Westerbacka J., Vehkavaara S., Häkkinen A., Olofsson S.O., Yki-Järvinen H. (2006). Overproduction of large VLDL particles is driven by increased liver fat content in man. Diabetologia.

[49] Kawano Y., Cohen D.E. (2013). Mechanisms of hepatic triglyceride accumulation in non-alcoholic fatty liver disease. J. Gastroenterol..

[50] Fujita K., Nozaki Y., Wada K., Yoneda M., Fujimoto Y., Fujitake M., Endo H., Takahashi H., Inamori M., Kobayashi N. (2009). Dysfunctional very-low-density lipoprotein synthesis and release is a key factor in nonalcoholic steatohepatitis pathogenesis. Hepatology.

[51] Cherubini A., Casirati E., Tomasi M., Valenti L. (2021). PNPLA3 as a therapeutic target for fatty liver disease: The evidence to date. Expert Opin. Ther. Targets.

[52] Zhang C., Ji J., Du X., Zhang L., Song Y., Wang Y., Jiang Y., Li K., Chang T. (2024). Atg5-deficient mesenchymal stem cells protect against non-alcoholic fatty liver by accelerating hepatocyte growth factor secretion. Cell Commun. Signal..

[53] Xia H., Zhu X., Zhang X., Jiang H., Li B., Wang Z., Li D., Jin Y. (2019). Alpha-naphthoflavone attenuates non-alcoholic fatty liver disease in oleic acid-treated HepG2 hepatocytes and in high fat diet-fed mice. Biomed. Pharmacother..

[54] Zheng Y., Wang S., Wu J., Wang Y. (2023). Mitochondrial metabolic dysfunction and non-alcoholic fatty liver disease: New insights from pathogenic mechanisms to clinically targeted therapy. J. Transl. Med..

[55] Sanders F.W., Griffin J.L. (2016). De novo lipogenesis in the liver in health and disease: More than just a shunting yard for glucose. Biol. Rev. Camb. Philos. Soc..

[56] Rector R.S., Thyfault J.P., Uptergrove G.M., Morris E.M., Naples S.P., Borengasser S.J., Mikus C.R., Laye M.J., Laughlin M.H., Booth F.W. (2010). Mitochondrial dysfunction precedes insulin resistance and hepatic steatosis and contributes to the natural history of non-alcoholic fatty liver disease in an obese rodent model. J. Hepatol..

[57] Navarro C.D.C., Figueira T.R., Francisco A., Dal’Bo G.A., Ronchi J.A., Rovani J.C., Escanhoela C.A.F., Oliveira H.C.F., Castilho R.F., Vercesi A.E. (2017). Redox imbalance due to the loss of mitochondrial NAD(P)-transhydrogenase markedly aggravates high fat diet-induced fatty liver disease in mice. Free Radic. Biol. Med..

[58] Meex R.C.R., Blaak E.E. (2021). Mitochondrial dysfunction is a key pathway that links saturated fat intake to the development and progression of NAFLD. Mol. Nutr. Food Res..

[59] Massart J., Begriche K., Hartman J.H., Fromenty B. (2022). Role of mitochondrial cytochrome P450 2E1 in healthy and diseased liver. Cells.

[60] Chi X., Yan R., Zhang J., Zhang G., Zhang Y., Hao M., Zhang Z., Fan P., Dong Y., Yang Y. (2020). A neutralizing human antibody binds to the N-terminal domain of the Spike protein of SARS-CoV-2. Science.

[61] Zeng C., Chen M. (2022). Progress in Nonalcoholic Fatty Liver Disease: SIRT Family Regulates Mitochondrial Biogenesis. Biomolecules.

[62] Chimienti G., Orlando A., Russo F., D’Attoma B., Aragno M., Aimaretti E., Lezza A.M.S., Pesce V. (2021). The Mitochondrial Trigger in an Animal Model of Nonalcoholic Fatty Liver Disease. Genes.

[63] Gancheva S., Kahl S., Pesta D., Mastrototaro L., Dewidar B., Strassburger K., Sabah E., Esposito I., Weiß J., Sarabhai T. (2022). Impaired Hepatic Mitochondrial Capacity in Nonalcoholic Steatohepatitis Associated with Type 2 Diabetes. Diabetes Care.

[64] Moore M.P., Cunningham R.P., Meers G.M., Johnson S.A., Wheeler A.A., Ganga R.R., Spencer N.M., Pitt J.B., Diaz-Arias A., Swi A.I.A. (2022). Compromised hepatic mitochondrial fatty acid oxidation and reduced markers of mitochondrial turnover in human NAFLD. Hepatology.

[65] Pirola C.J., Gianotti T.F., Burgueno A.L., Rey-Funes M., Loidl C.F., Mallardi P., Martino J.S., Castano G.O., Sookoian S. (2013). Epigenetic modification of liver mitochondrial DNA is associated with histological severity of nonalcoholic fatty liver disease. Gut.

[66] Hou J., Zhang J., Cui P., Zhou Y., Liu C., Wu X., Ji Y., Wang S., Cheng B., Ye H. (2021). TREM2 sustains macrophage-hepatocyte metabolic coordination in nonalcoholic fatty liver disease and sepsis. J. Clin. Investig..

[67] Mao H., Chen W., Chen L., Li L. (2022). Potential role of mitochondria-associated endoplasmic reticulum membrane proteins in diseases. Biochem. Pharmacol..

[68] Lazarou M., Siter D.A., Kane L.A., Sarraf S.A., Wang C., Burman J.L., Sideris D.P., Fogel A.I., Youle R.J. (2015). The ubiquitin kinase PINK1 recruits autophagy receptors to induce mitophagy. Nature.

[69] Vranas M., Lu Y., Rasool S., Croteau N., Krett J.D., Sauve V., Gehring K., Fon E.A., Durcan T.M., Trempe J.F. (2022). Selective localization of Mfn2 near PINK1 enables its preferential ubiquitination by parkin on mitochondria. Open Biol..

[70] Glick D., Zhang W., Beaton M., Marsboom G., Gruber M., Simon M.C., Hart J., Dorn G.W., Brady M.J., Macleod K.F. (2012). BNip3 regulates mitochondrial function and lipid metabolism in the liver. Mol. Cell. Biol..

[71] Suomalainen A., Battersby B.J. (2018). Mitochondrial diseases: The contribution of organelle stress responses to pathology. Nat. Rev. Mol. Cell Biol..

[72] Zhou T., Chang L., Luo Y., Zhou Y., Zhang J. (2019). Mst1 inhibition attenuates non-alcoholic fatty liver disease via reversing Parkin-related mitophagy. Redox Biol..

[73] Li R., Toan S., Zhou H. (2020). Role of mitochondrial quality control in the pathogenesis of nonalcoholic fatty liver disease. Aging.

[74] Cioffi F., Giacco A., Petito G., de Matteis R., Senese R., Lombardi A., de Lange P., Moreno M., Goglia F., Lanni A. (2022). Altered mitochondrial quality control in rats with metabolic dysfunction-associated fatty liver disease (MAFLD) induced by high-fat feeding. Genes.

[75] Roca-Portoles A., Tait S.W.G. (2021). Mitochondrial quality control: From molecule to organelle. Cell. Mol. Life Sci..

[76] Krishnasamy Y., Gooz M., Li L., Lemasters J.J., Zhong Z. (2019). Role of mitochondrial depolarization and disrupted mitochondrial homeostasis in nonalcoholic steatohepatitis and fibrosis in mice. Int. J. Physiol. Pathophysiol. Pharmacol..

[77] Sheldon R.D., Meers G., Morris E.M., Linden M.A., Cunningham R.P., Ibdah J.A., Thyfault J.P., Laughlin M.H., Rector R.S. (2019). eNOS deletion impairs mitochondrial quality control and exacerbates Western diet-induced NASH. Am. J. Physiol.-Endocrinol. Metab..

[78] Sanyal A.J., Chalasani N., Kowdley K.V., McCullough A., Diehl A.M., Bass N.M., Neuschwander-Tetri B.A., Lavine J.E., Tonascia J., Unalp A. (2010). Pioglitazone, vitamin E, or placebo for nonalcoholic steatohepatitis. N. Engl. J. Med..

[79] Gastaldelli A., Cusi K. (2019). From NASH to diabetes and from diabetes to NASH: Mechanisms and treatment options. JHEP Rep..

[80] Armstrong M.J., Gaunt P., Aithal G.P., Barton D., Hull D., Parker R., Hazlehurst J.M., Guo K., Abouda G., LEAN Trial Team (2016). Liraglutide safety and efficacy in patients with non-alcoholic steatohepatitis (LEAN): A multicentre, double-blind, randomised, placebo-controlled phase 2 study. Lancet.

[81] Paternostro R., Trauner M. (2022). Current treatment of non-alcoholic fatty liver disease. J. Intern. Med..

[82] Neuschwander-Tetri B.A., Loomba R., Sanyal A.J., Lavine J.E., Van Natta M.L., Abdelmalek M.F., Chalasani N., Dasarathy S., Diehl A.M., Hameed B. (2015). Farnesoid X nuclear receptor ligand obeticholic acid for non-cirrhotic, non-alcoholic steatohepatitis (FLINT): A multicentre, randomised, placebo-controlled trial. Lancet.

[83] Younossi Z.M., Ratziu V., Loomba R., Rinella M., Anstee Q.M., Goodman Z., Bedossa P., Geier A., Beckebaum S., Newsome P.N. (2019). Obeticholic acid for the treatment of non-alcoholic steatohepatitis: Interim analysis from a multicentre, randomised, placebo-controlled phase 3 trial. Lancet.

[84] Feillet-Coudray C., Fouret G., Ebabe Elle R., Rieusset J., Bonafos B., Chabi B., Crouzier D., Zarkovic K., Zarkovic N., Ramos J. (2014). The mitochondrial-targeted antioxidant MitoQ ameliorates metabolic syndrome features in obesogenic diet-fed rats better than apocynin or Allopurinol. Free Radic. Res..

[85] Rokitskaya T.I., Klishin S.S., Severina I.I., Skulachev V.P., Antonenko Y.N. (2008). Kinetic analysis of permeation of mitochondria-targeted antioxidants across bilayer lipid membranes. J. Membr. Biol..

[86] Zhang J., Xiang H., Liu J., Chen Y., He R.R., Liu B. (2020). Mitochondrial sirtuin 3: New emerging biological function and therapeutic target. Theranostics.

[87] Zhang T., Liu J., Shen S., Tong Q., Ma X., Lin L. (2020). SIRT3 promotes lipophagy and chaperon-mediated autophagy to protect hepatocytes against lipotoxicity. Cell Death Differ..

[88] Fu A., Shi X., Zhang H., Fu B. (2017). Mitotherapy for fatty liver by intravenous administration of exogenous mitochondria in male mice. Front. Pharmacol..

[89] Ajith T.A. (2018). Role of mitochondria and mitochondria-targeted agents in non-alcoholic fatty liver disease. Clin. Exp. Pharmacol. Physiol..

[90] Bojic L.A., Huff M.W. (2013). Peroxisome proliferator-activated receptor delta: A multifaceted metabolic player. Curr. Opin. Lipidol..

[91] Tong W., Ju L., Qiu M., Xie Q., Chen Y., Shen W., Sun W., Wang W., Tian J. (2016). Liraglutide ameliorates non-alcoholic fatty liver disease by enhancing mitochondrial architecture and promoting autophagy through the SIRT1/SIRT3-FOXO3a pathway. Hepatol. Res..

[92] Sinha R.A., Bruinstroop E., Singh B.K., Yen P.M. (2019). Nonalcoholic Fatty Liver Disease and Hypercholesterolemia: Roles of Thyroid Hormones, Metabolites, and Agonists. Thyroid.

[93] Zucchi R. (2020). Thyroid Hormone Analogues: An Update. Thyroid.

[94] Coppola M., Glinni D., Moreno M., Cioffi F., Silvestri E., Goglia F. (2014). Thyroid hormone analogues and derivatives: Actions in fatty liver. World J. Hepatol..

[95] Dufour J.F., Caussy C., Loomba R. (2019). Bariatric surgery in patients with non–alcoholic fatty liver disease—From pathophysiology to clinical effects. World J. Hepatol..

[96] Lee Y., Doumouras A.G., Yu J., Brar K., Banfield L., Gmora S., Anvari M., Hong D. (2019). Complete Resolution of Nonalcoholic Fatty Liver Disease After Bariatric Surgery: A Systematic Review and Meta-analysis. Clin. Gastroenterol. Hepatol..

[97] Bird S.R., Hawley J.A. (2017). Update on the effects of physical activity on insulin sensitivity in humans. BMJ Open Sport Exerc. Med..

[98] Steckhan N., Hohmann C.D., Kessler C., Dobos G., Michalsen A., Cramer H. (2016). Effects of different dietary approaches on inflammatory markers in patients with metabolic syndrome: A systematic review and meta-analysis. Nutrition.

[99] Rector R.S., Thyfault J.P., Morris R.T., Laye M.J., Borengasse S.J., Booth F.W., Ibdah J.A. (2008). Daily exercise increases hepatic fatty acid oxidation and prevents steatosis in Otsuka Long-Evans Tokushima fatty rats. Am. J. Physiol. Gastrointest. Liver Physiol..

[100] Fernandes M.S.S., Silva L., Kubrusly M.S., Lima T., Muller C.R., Americo A.L.V., Fernandes M.P., Cogliati B., Stefano J.T., Lagranha C.J. (2020). Aerobic exercise training exerts beneficial effects upon oxidative metabolism and non-enzymatic antioxidant defense in the liver of Leptin Deficiency mice. Front. Endocrinol..

[101] Misciagna G., Del Pilar Díaz M., Caramia D.V., Bonfiglio C., Franco I., Noviello M.R., Chiloiro M., Abbrescia D.I., Mirizzi A., Tanzi M. (2017). Effect of a Low Glycemic Index Mediterranean Diet on Non-Alcoholic Fatty Liver Disease. A Randomized Controlled Clinici Trial. J. Nutr. Health Aging.

[102] Katsagoni C.N., Georgoulis M., Papatheodoridis G.V., Fragopoulou E., Ioannidou P., Papageorgiou M., Alexopoulou A., Papadopoulos N., Deutsch M., Kontogianni M.D. (2017). Associations between lifestyle characteristics and the presence of non-alcoholic fatty liver disease: A case-control study. Metab. Syndr. Relat. Disord..

[103] Keys A. (1995). Mediterranean diet and public health: Personal reflections. Am. J. Clin. Nutr..

[104] Urquiaga I., Echeverría G., Dussaillant C., Rigotti A. (2017). Origen, components y posibles mecanismos de acción de la dieta mediterránea [Origin, components and mechanisms of action of the Mediterranean diet]. Rev. Med. Chil..

[105] Simopoulos A.P. (2001). The Mediterranean diets: What is so special about the diet of Greece? The scientific evidence. J. Nutr..

[106] Bach-Faig A., Berry E.M., Lairon D., Reguant J., Trichopoulou A., Dernini S., Medina F.X., Battino M., Belahsen R., Miranda G. (2011). Mediterranean diet pyramid today. Science and cultural updates. Public Health Nutr..

[107] Ross A.B., van der Kamp J.W., King R., Lê K.A., Mejborn H., Seal C.J., Thielecke F. (2017). Perspective: A Definition for Whole-Grain Food Products—Recommendations from the Healthgrain Forum. Adv. Nutr..

[108] Cavazos A., De Mejia E.G. (2013). Identification of Bioactive Peptides from Cereal Storage Proteins and Their Potential Role in Prevention of Chronic Diseases. Compr. Rev. Food Sci. Food Saf..

[109] Brites C., Braz N., Mateus M., Bernardes J.P., Freitas A. (2015). Cereals in the context of the Mediterranean Diet. Dimensions of Mediterranean Diet: World Cultural Heritage.

[110] De Jong N., Hoendervangers C.T., Bleeker J.K., Ocké M.C. (2004). The opinion of Dutch dietitians about functional foods. J. Hum. Nutr. Diet..

[111] Laddomada B., Caretto S., Mita G. (2015). Wheat bran phenolic acids: Bioavailability and stability in whole wheat-based foods. Molecules.

[112] Huang T., Xu M., Lee A., Cho S., Qi L. (2015). Consumption of whole grains and cereal fiber and total and cause-specific mortality: Prospective analysis of 367,442 individuals. BMC Med..

[113] Liaqat H., Kim K.J., Park S.Y., Jung S.K., Park S.H., Lim S., Kim J.Y. (2021). Antioxidant effect of wheat germ extracts and their antilipidemic effect in palmitic acid-induced steatosis in HepG2 and 3T3-L1 cells. Foods.

[114] Bechthold A., Boeing H., Schwedhelm C., Hoffmann G., Knuppel S., Iqbal K., Schwingshackl L. (2019). Food groups and risk of coronary heart disease, stroke and heart failure: A systematic review and dose response meta-analysis of prospective studies. Crit. Rev. Food Sci. Nutr..

[115] Schlesinger S., Neuenschwander M., Schwedhelm C., Hoffmann G., Bechthold A., Boeing H., Schwingshackl L. (2019). Food Groups and Risk of Overweight, Obesity, and Weight Gain: A Systematic Review and Dose-Response Meta-Analysis of Prospective Studies. Adv. Nutr..

[116] Naureen Z., Dhuli K., Donato K., Aquilanti B., Velluti V., Matera G., Iaconelli A., Bertelli M. (2022). Foods of the Mediterranean diet: Tomato, olives, chili pepper, wheat flour and wheat germ. J. Prev. Med. Hyg..

[117] Sun Q., Du M., Navarre D.A., Zhu M. (2021). Effect of Cooking Methods on Bioactivity of Polyphenols in Purple Potatoes. Antioxidants.

[118] Vargha S., Igual M., Miraballes M., Gámbaro A., García-Segovia P., Martínez-Monzó J. (2024). Influence of Cooking Technique on Bioaccessibility of Bioactive Compounds in Vegetable Lentil Soup. Foods.

[119] González-Coria J., Mesirca-Prevedello C., Lozano-Castellón J., Casadei E., Valli E., López-Yerena A., Jaime-Rodríguez C., Pinto D., Illan M., Torrado X. (2024). Chemometric study on the effect of cooking on bioactive compounds in tomato pomace enriched sauces. Npj Sci. Food.

[120] Delgado A., Vaz-Almeida M., Parisi S. (2017). Chemistry of the Mediterranean Diet.

[121] Schwingshackl L., Schwedhelm C., Galbete C., Hoffmann G. (2017). Adherence to Mediterranean diet and risk of cancer: An updated systematic review and meta-analysis. Nutrients.

[122] Schwingshackl L., Hoffmann G., Lampousi A.M., Knuppel S., Iqbal K., Schwedhelm C., Bechthold A., Schlesinger S., Boeing H. (2017). Food groups and risk of type 2 diabetes mellitus: A systematic review and meta-analysis of prospective studies. Eur. J. Epidemiol..

[123] Tan H., Thomas-Ahner J.M., Grainger E.M., Wan L., Francis D.M., Schwartz S.J., Clinton S.K. (2010). Tomato-based food products for prostate cancer prevention: What have we learned?. Cancer Metastasis Rev..

[124] Chaudhary P., Sharma A., Singh B., Nagpal A.K. (2018). Bioactivities of phytochemicals present in tomato. J. Food Sci. Technol..

[125] Martí R., Leiva-Brondo M., Lahoz I., Campillo C., Cebolla Cornejo J., Roselló S. (2018). Polyphenol and l-ascorbic acid content in tomato as influenced by high lycopene genotypes and organic farming at different environments. Food Chem..

[126] Viuda-Martos M., Sanchez-Zapata E., Sayas-Barberá E., Sendra E., Pérez-Álvarez J.A., Fernández-López J. (2014). Tomato and tomato byproducts. Human health benefits of lycopene and its application to meat products: A review. Crit. Rev. Food Sci. Nutr..

[127] Abenavoli L., Procopio A.C., Paravati M.R., Costa G., Milić N., Alcaro S., Luzza F. (2022). Mediterranean Diet: The Beneficial Effects of Lycopene in Non-Alcoholic Fatty Liver Disease. J. Clin. Med..

[128] Ferreira H., Pinto E., Vasconcelos M.W. (2021). Legumes as a Cornerstone of the Transition Toward More Sustainable Agri-Food Systems and Diets in Europe. Front. Sustain. Food Syst..

[129] Davis C., Bryan J., Hodgson J., Murphy K. (2015). Definition of the Mediterranean Diet: A Literature Review. Nutrients.

[130] Hernández-López I., Ortiz-Solà J., Alamprese C., Barros L., Shelef O., Basheer L., Rivera A., Abadias M., Aguiló-Aguayo I. (2022). Valorization of Local Legumes and Nuts as Key Components of the Mediterranean Diet. Foods.

[131] Guasch-Ferré M., Willett W.C. (2021). The Mediterranean diet and health: A comprehensive overview. J. Intern. Med..

[132] Sobczak P., Grochowicz J., Łusiak P., Zukiewicz-Sobczak W. (2023). Development of Alternative Protein Sources in Terms of a Sustainable System. Sustainability.

[133] Van Loon M.P., Alimagham S., Pronk A., Fodor N., Ion V., Kryvoshein O., Kryvobok O., Marrou H., Mihail R., Mínguez M.I. (2023). Grain legume production in Europe for food, feed and meat-substitution. Glob. Food Secur..

[134] Dominguez L.J., Barbagallo M., Preedy V.R., Watson R.R. (2020). Dietary Fiber Intake and the Mediterranean Population. The Mediterranean Diet.

[135] Conti M.V., Guzzetti L., Panzeri D., De Giuseppe R., Coccetti P., Labra M., Cena H. (2021). Bioactive compounds in legumes: Implications for sustainable nutrition and health in the elderly population. Trends Food Sci. Technol..

[136] Salehi B., Quispe C., Sharifi-Rad J., Cruz-Martins N., Nigam M., Mishra A.P., Konovalov D.A., Orobinskaya V., Abu-Reidah I.M., Zam W. (2021). Phytosterols: From Preclinical Evidence to Potential Clinical Applications. Front. Pharmacol..

[137] Frasinariu O., Serban R., Trandafir L.M., Miron I., Starcea M., Vasiliu I., Alisi A., Temneanu O.R. (2022). The Role of Phytosterols in Nonalcoholic Fatty Liver Disease. Nutrients.

[138] Mendivil C.O. (2021). Fish Consumption: A Review of Its Effects on Metabolic and Hormonal Health. Nutr. Metab. Insights.

[139] Molina-Vega M., Gómez-Pérez A.M., Tinahones F.J., Preedy V.R., Watson R.R. (2020). Fish in the Mediterranean diet. the Mediterranean Diet.

[140] Adeva-Andany M.M., Calvo-Castro I., Fernández-Fernández C., Donapetry-García C., Pedre-Piñeiro A.M. (2017). Significance of l-carnitine for human health. IUBMB Life.

[141] Dijck-Brouwer D.A.J., Muskiet F.A.J., Verheesen R.H., Schaafsma G., Schaafsma A., Geurts J.M.W. (2022). Thyroidal and Extrathyroidal Requirements for Iodine and Selenium: A Combined Evolutionary and (Patho)Physiological Approach. Nutrients.

[142] Cioffi F., Giacco A., Goglia F., Silvestri E. (2022). Bioenergetic Aspects of Mitochondrial Actions of Thyroid Hormones. Cells.

[143] Hatziagelaki E., Paschou S.A., Schön M., Psaltopoulou T., Roden M. (2022). NAFLD and thyroid function: Pathophysiological and therapeutic considerations. Trends Endocrinol. Metab..

[144] Abdallah A.A.M., Abdelrahman M.M., Attia H.M.A.S., Hafez A., Anwar Rashed S., Amin Y.A., Hemdan S.B. (2022). Decreased Serum zinc, selenium, and vitamin E as possible risk factors of hepatic fibrosis in non-alcoholic fatty liver disease. Nutr. Health.

[145] Wang Y., Liu B., Wu P., Chu Y., Gui S., Zheng Y., Chen X. (2022). Dietary Selenium Alleviated Mouse Liver Oxidative Stress and NAFLD Induced by Obesity by Regulating the KEAP1/NRF2 Pathway. Antioxidants.

[146] Guixà-González R., Javanainen M., Gómez-Soler M., Cordobilla B., Domingo J.C., Sanz F., Pastor M., Ciruela F., Martinez-Seara H., Selent J. (2016). Membrane omega-3 fatty acids modulate the oligomerisation kinetics of adenosine A2A and dopamine D2 receptors. Sci. Rep..

[147] Shahidi F., Ambigaipalan P. (2018). Omega-3 Polyunsaturated Fatty Acids and Their Health Benefits. Annu. Rev. Food Sci. Technol..

[148] Djuricic I., Calder P.C. (2021). Beneficial Outcomes of Omega-6 and Omega-3 Polyunsaturated Fatty Acids on Human Health: An Update for 2021. Nutrients.

[149] Musa-Veloso K., Venditti C., Lee H.Y., Darch M., Floyd S., West S., Simon R. (2018). Systematic review and meta-analysis of controlled intervention studies on the effectiveness of long-chain omega-3 fatty acids in patients with nonalcoholic fatty liver disease. Nutr. Rev..

[150] Mantzioris E., Muhlhausler B.S., Villani A. (2022). Impact of the Mediterranean Dietary pattern on n-3 fatty acid tissue levels-A systematic review. Prostaglandins Leukot. Essent. Fat. Acids.

[151] Schwingshackl L., Schlesinger S., Devleesschauwer B., Hoffmann G., Bechthold A., Schwedhelm C., Iqbal K., Knüppel S., Boeing H. (2018). Generating the evidence for risk reduction: A contribution to the future of food based dietary guidelines. Proc. Nutr. Soc..

[152] Harris W., Miller M., Tighe A.P., Davidson M.H., Schaefer E.J. (2008). Omega-3 fatty acids and coronary heart disease risk: Clinical and mechanistic perspectives. Atherosclerosis.

[153] Ros E., Singh A., O’Keefe J.H. (2021). Nuts: Natural Pleiotropic Nutraceuticals. Nutrients.

[154] Agricultural Research Service, US Department of Agriculture, Agricultural Research Service (2019). Food Data Central.

[155] Kornsteiner-Krenn M., Wagner K.H., Elmadfa I. (2013). Phytosterol content and fatty acid pattern of ten different nut types. Int. J. Vitam. Nutr. Res..

[156] Neveu V., Perez-Jiménez J., Vos F., Crespy V., du Chaffaut L., Mennen L., Knox C., Eisner R., Cruz J., Wishart D. (2010). Phenol-Explorer: An online comprehensive database on polyphenol contents in foods. Database.

[157] Del Gobbo L.C., Falk M.C., Feldman R., Lewis K., Mozaffarian D. (2015). Are Phytosterols Responsible for the Low-Density Lipoprotein-Lowering Effects of Tree Nuts?: A Systematic Review and Meta-Analysis. J. Am. Coll. Cardiol..

[158] Karppanen H., Karppanen P., Mervaala E. (2005). Why and how to implement sodium, potassium, calcium, and magnesium changes in food items and diets?. J. Hum. Hypertens..

[159] Jackson C.L., Hu F.B. (2014). Long-term associations of nut consumption with body weight and obesity. Am. J. Clin. Nutr..

[160] Plaz Torres M.C., Bodini G., Furnari M., Marabotto E., Zentilin P., Giannini E.G. (2020). Nuts and Non-Alcoholic Fatty Liver Disease: Are Nuts Safe for Patients with Fatty Liver Disease?. Nutrients.

[161] Pan L., Sui J., Xu Y., Zhao Q. (2023). Effect of Nut Consumption on Nonalcoholic Fatty Liver Disease: A Systematic Review and Meta-Analysis. Nutrients.

[162] Tan S.Y., Georgousopoulou E.N., Cardoso B.R., Daly R.M., George E.S. (2021). Associations between nut intake, cognitive function and non-alcoholic fatty liver disease (NAFLD) in older adults in the United States: NHANES 2011-14. BMC Geriatr..

[163] AOCS. https://www.aocs.org/publications-resources/resource-library/lipid-library/.

[164] Lopez S., Bermudez B., Montserrat-de la Paz S., Jaramillo S., Varela L.M., Ortega-Gomez A., Abia R., Muriana F.J. (2014). Membrane composition and dynamics: A target of bioactive virgin olive oil constituents. Biochim. Biophys. Acta.

[165] Corona G., Spencer J.P., Dessì M.A. (2009). Extra virgin olive oil phenolics: Absorption, metabolism, and biological activities in the GI tract. Toxicol. Ind. Health.

[166] Flynn M.M., Tierney A., Itsiopoulos C. (2023). Is Extra Virgin Olive Oil the Critical Ingredient Driving the Health Benefits of a Mediterranean Diet? A Narrative Review. Nutrients.

[167] Pirozzi C., Lama A., Simeoli R., Paciello O., Pagano T.B., Mollica M.P., Di Guida F., Russo R., Magliocca S., Canani R.B. (2016). Hydroxytyrosol prevents metabolic impairment reducing hepatic inflammation and restoring duodenal integrity in a rat model of NAFLD. J. Nutr. Biochem..

[168] Zhao B., Ma Y., Xu Z., Wang J., Wang F., Wang D., Pan S., Wu Y., Pan H., Xu D. (2014). Hydroxytyrosol, a natural molecule from olive oil, suppresses the growth of human hepatocellular carcinoma cells via inactivating AKT and nuclear factor kappa B pathways. Cancer Lett..

[169] Lee D.H., Han D.H., Nam K.T., Park J.S., Kim S.H., Lee M., Kim G., Min B.S., Cha B.S., Lee Y.S. (2016). Ezetimibe, an NPC1L1 inhibitor, is a potent Nrf2 activator that protects mice from diet-induced nonalcoholic steatohepatitis. Free Radic. Biol. Med..

[170] Martín M.A., Ramos S., Granado-Serrano A.B., Rodríguez Ramiro I., Trujillo M., Bravo L., Goya L. (2010). Hydroxytyrosol in duces antioxidant/detoxificant enzymes and Nrf2 translocation via extracelular regulated kinases and phosphatidylinositol-3 kinase/protein kinase B pathways in HepG2 cells. Mol. Nutr. Food Res..

[171] Ruiz-Gutiérrez V., De La Puerta R., Catala A. (2001). The effect of tyrosol, hydroxytyrosol and oleuropein on the non-enzymic lipid per oxidation of rat liver microsomes. Mol. Cell. Biochem..

[172] Schirone L., Overi D., Carpino G., Carnevale R., De Falco E., Nocella C., D’Amico A., Bartimoccia S., Cammisotto V., Castellani V. (2024). Oleuropein, a Component of Extra Virgin Olive Oil, Improves Liver Steatosis and Lobular Inflammation by Lipopolysaccharides-TLR4 Axis Downregulation. Int. J. Mol. Sci..

[173] Valenzuela R., Videla L.A. (2020). Impact of the Co-Administration of N-3 Fatty Acids and Olive Oil Components in Preclinical Nonalcoholic Fatty Liver Disease Models: A Mechanistic View. Nutrients.

[174] Schwingshackl L., Hoffmann G. (2014). Monounsaturated fatty acids, olive oil and health status: A systematic review and meta-analysis of cohort studies. Lipids Health Dis..

[175] Schwingshackl L., Missbach B., Konig J., Hoffmann G. (2015). Adherence to a Mediterranean diet and risk of diabetes: A systematic review and meta-analysis. Public Health Nutr..

[176] Tedesco C.C., Bonfiglio C., Notarnicola M., Rendina M., Castellaneta A., Di Leo A., Giannelli G., Fontana L. (2023). High Extra Virgin Olive Oil Consumption Is Linked to a Lower Prevalence of NAFLD with a Prominent Effect in Obese Subjects: Results from the MICOL Study. Nutrients.

[177] Pintó X., Fanlo-Maresma M., Corbella E., Corbella X., Mitjavila M.T., Moreno J.J., Casas R., Estruch R., Corella D., Bulló M. (2019). A Mediterranean Diet Rich in Extra-Virgin Olive Oil Is Associated with a Reduced Prevalence of Nonalcoholic Fatty Liver Disease in Older Individuals at High Cardiovascular Risk. J. Nutr..

[178] Paniagua J., Gallego de la Sacristana A., Romero I., Vidal-Puig A., Latre J., Sanchez E., Perez-Martinez P., Lopez-Miranda J., Perez-Jimenez F. (2007). Monounsaturated Fat—Rich Diet Prevents Expression Induced by a Carbohydrate—Rich Diet in Insulin-Resistant Subjects. Diabetes Care.

[179] Jiménez-Sánchez A., Martínez-Ortega A.J., Remón-Ruiz P.J., Piñar-Gutiérrez A., Pereira-Cunill J.L., García-Luna P.P. (2022). Therapeutic Properties and Use of Extra Virgin Olive Oil in Clinical Nutrition: A Narrative Review and Literature Update. Nutrients.

[180] Berrougui H., Ikhlef S., Khalil A. (2015). Extra Virgin Olive Oil Polyphenols Promote Cholesterol Efflux and Improve HDL Functionality. Evid.-Based Complement. Altern. Med..

[181] Moreno-Luna R., Muñoz-Hernandez R., Miranda M.L., Costa A.F., Jimenez-Jimenez L., Vallejo-Vaz A.J., Muriana F.J.G., Villar J., Stiefel P. (2012). Olive Oil Polyphenols Decrease Blood Pressure and Improve Endothelial Function in Young Women with Mild Hypertension. Am. J. Hypertens..

[182] Che X., Li L., Liu X., Luo R., Liao G., Li L., Liu J., Cheng J., Lu Y., Chen Y. (2018). Oleic acid protects saturated fatty acid mediated lipotoxicity in hepatocytes and rat of non-alcoholic steatohepatitis. Life Sci..

[183] Liu X., Li X., Su S., Yuan Y., Liu W., Zhu M., Zheng Q., Zeng X., Fu F., Lu Y. (2023). Oleic acid improves hepatic lipotoxicity injury by alleviating autophagy dysfunction. Exp. Cell Res..

[184] Mäkelä T.N.K., Tuomainen T.P., Hantunen S., Virtanen J.K. (2022). Associations of Serum n–3 and n–6 Polyunsaturated Fatty Acids with Prevalence and Incidence of Nonalcoholic Fatty Liver Disease. Am. J. Clin. Nutr..

[185] Zhao H., Yang A., Mao L., Quan Y., Cui J., Sun Y. (2020). Association Between Dietary Fiber Intake and Non-Alcoholic Fatty Liver Disease in Adults. Front. Nutr..

[186] Zhu Y., Yang H., Zhang Y., Rao S., Mo Y., Zhang H., Liang S., Zhang Z., Yang W. (2023). Dietary Fiber Intake and Non-Alcoholic Fatty Liver Disease: The Mediating Role of Obesity. Front. Public Health.

[187] Musazadeh V., Karimi A., Malekahmadi M., Ahrabi S.S., Dehghan P. (2023). Omega-3 polyunsaturated fatty acids in the treatment of non-alcoholic fatty liver disease: An umbrella systematic review and meta-analysis. Clin. Exp. Pharmacol. Physiol..

[188] Spooner M.H., Jump D.B. (2023). Nonalcoholic Fatty Liver Disease and Omega-3 Fatty Acids: Mechanisms and Clinical Use. Annu. Rev. Nutr..

[189] Yang J., Fernández-Galilea M., Martínez-Fernández L., González-Muniesa P., Pérez-Chávez A., Martínez J.A., Moreno-Aliaga M.J. (2019). Oxidative Stress and Non-Alcoholic Fatty Liver Disease: Effects of Omega-3 Fatty Acid Supplementation. Nutrients.

[190] Anania C., Perla F.M., Olivero F., Pacifico L., Chiesa C. (2018). Mediterranean diet and nonalcoholic fatty liver disease. World J. Gastroenterol..

[191] Vinelli V., Biscotti P., Martini D., Del Bo’ C., Marino M., Meroño T., Nikoloudaki O., Calabrese F.M., Turroni S., Taverniti V. (2022). Effects of Dietary Fibers on Short-Chain Fatty Acids and Gut Microbiota Composition in Healthy Adults: A Systematic Review. Nutrients.

[192] So D., Whelan K., Rossi M., Morrison M., Holtmann G., Kelly J.T., Shanahan E.R., Staudacher H.M., Campbell K.L. (2018). Dietary Fiber Intervention on Gut Microbiota Composition in Healthy Adults: A Systematic Review and Meta-Analysis. Am. J. Clin. Nutr..

[193] Zhang S., Zhao J., Xie F., He H., Johnston L.J., Dai X., Wu C., Ma X. (2021). Dietary fiber-derived short-chain fatty acids: A potential therapeutic target to alleviate obesity-related nonalcoholic fatty liver disease. Obes. Rev..

[194] Park M., Yoo J.H., Lee Y.S., Lee H.J. (2019). *Lonicera caerulea* Extract Attenuates Non-Alcoholic Fatty Liver Disease in Free Fatty Acid-Induced HepG2 Hepatocytes and in High Fat Diet-Fed Mice. Nutrients.

[195] Zou W., Zhang C., Gu X., Li X., Zhu H. (2021). Metformin in Combination with Malvidin Prevents Progression of Non-Alcoholic Fatty Liver Disease via Improving Lipid and Glucose Metabolisms, and Inhibiting Inflammation in Type 2 Diabetes Rats. Drug Des. Dev. Ther..

[196] Afshari H., Noori S., Zarghi A. (2023). A novel combination of metformin and resveratrol alleviates hepatic steatosis by activating autophagy through the cAMP/AMPK/SIRT1 signaling pathway. Naunyn-Schmiedeberg’s Arch. Pharmacol..

[197] Hwang Y.P., Kim H.G., Choi J.H., Do M.T., Tran T.P., Chun H.K., Chung Y.C., Jeong T.C., Jeong H.G. (2013). 3-Caffeoyl, 4-Dihydrocaffeoylquinic Acid from Salicornia Herbacea Attenuates High Glucose-Induced Hepatic Lipogenesis in Human HepG2 Cells through Activation of the Liver Kinase B1 and Silent Information Regulator T1/AMPK-Dependent Pathway. Mol. Nutr. Food Res..

[198] Yang K., Chen J., Zhang T., Yuan X., Ge A., Wang S., Xu H., Zeng L., Ge J. (2022). Efficacy and safety of dietary polyphenol supplementation in the treatment of non-alcoholic fatty liver disease: A systematic review and meta-analysis. Front. Immunol..

[199] Baratta F., Pastori D., Bartimoccia S., Cammisotto V., Cocomello N., Colantoni A., Nocella C., Carnevale R., Ferro D., Angelico F. (2020). Poor Adherence to Mediterranean Diet and Serum Lipopolysaccharide are Associated with Oxidative Stress in Patients with Non-Alcoholic Fatty Liver Disease. Nutrients.

[200] Delli Bovi A.P., Marciano F., Mandato C., Siano M.A., Savoia M., Vajro P. (2021). Oxidative Stress in Non-Alcoholic Fatty Liver Disease. an Updated Mini Review. Front. Med..

[201] Clifford T., Acton J.P., Cocksedge S.P., Davies K.A.B., Bailey S.J. (2021). The Effect of Dietary Phytochemicals on Nuclear Factor Erythroid 2-Related Factor 2 (Nrf2) Activation: A Systematic Review of Human Intervention Trials. Mol. Biol. Rep..

[202] Sobhani M., Farzaei M.H., Kiani S., Khodarahmi R. (2021). Immunomodulatory; Anti-inflammatory/antioxidant Effects of Polyphenols: A Comparative Review on the Parental Compounds and Their Metabolites. Food Rev. Int..

[203] He X., Li Y., Deng X., Xiao X., Zeng J. (2023). Integrative evidence construction for resveratrol treatment of nonalcoholic fatty liver disease: Preclinical and clinical meta-analyses. Front Pharmacol..

[204] Yang J.W., Ji H.F. (2023). Phytosterols as bioactive food components against nonalcoholic fatty liver disease. Crit. Rev. Food Sci. Nutr..

[205] Xin Y., Li X., Zhu X., Lin X., Luo M., Xiao Y., Ruan Y., Guo H. (2023). Stigmasterol Protects Against Steatohepatitis Induced by High-Fat and High-Cholesterol Diet in Mice by Enhancing the Alternative Bile Acid Synthesis Pathway. J. Nutr..

[206] Abo-Zaid O.A., Moawed F.S., Ismail E.S., Farrag M.A. (2023). β-sitosterol attenuates high-fat diet-induced hepatic steatosis in rats by modulating lipid metabolism, inflammation and ER stress pathway. BMC Pharmacol. Toxicol..

[207] Gumede N.M., Lembede B.W., Brooksbank R.L., Erlwanger K.H., Chivandi E. (2020). β-Sitosterol Shows Potential to Protect Against the Development of High-Fructose Diet-Induced Metabolic Dysfunction in Female Rats. J. Med. Food.

[208] Han H., Xue T., Li J., Guo Y., Li X., Wang L., Pei L., Zheng M. (2022). Plant sterol ester of α-linolenic acid improved non-alcoholic fatty liver disease by attenuating endoplasmic reticulum stress-triggered apoptosis via activation of the AMPK. J. Nutr. Biochem..

[209] Han H., Li J., Tian L., Pei L., Zheng M. (2023). Through regulation of the SIRT1 pathway plant sterol ester of α-linolenic acid inhibits pyroptosis thereby attenuating the development of NASH in mice. J. Nutr. Biochem..

[210] Tauriainen M.M., Männistö V., Kaminska D., Vaittinen M., Kärjä V., Käkelä P., Venesmaa S., Gylling H., Pihlajamäki J. (2018). Serum, liver and bile sitosterol and sitostanol in obese patients with and without NAFLD. Biosci. Rep..

[211] Shahi M.M., Javanmardi M.A., Seyedian S.S., Haghighizadeh M.H. (2018). Effects of Phytosterol Supplementation on Serum Levels of Lipid Profiles, Liver Enzymes, Inflammatory Markers, Adiponectin, and Leptin in Patients Affected by Nonalcoholic Fatty Liver Disease: A Double-Blind, Placebo-Controlled, Randomized Clinical Trial. J. Am. Coll. Nutr..

[212] Chen D.L., Huang P.H., Chiang C.H., Leu H.B., Chen J.W., Lin S.J. (2015). Phytosterols increase circulating endothelial progenitor cells and insulin-like growth factor-1 levels in patients with nonalcoholic fatty liver disease: A randomized crossover study. J. Funct. Foods.

[213] Song L., Zhao X.G., Ouyang P.L., Guan Q., Yang L., Peng F., Du H., Yin F., Yan W., Yu W.J. (2020). Combined effect of *n*-3 fatty acids and phytosterol esters on alleviating hepatic steatosis in non-alcoholic fatty liver disease subjects: A double-blind placebo-controlled clinical trial. Br. J. Nutr..

[214] Ding X., Xu Y., Nie P., Zhong L., Feng L., Guan Q., Song L. (2022). Changes in the serum metabolomic profiles of subjects with NAFLD in response to n-3 PUFAs and phytosterol ester: A double-blind randomized controlled trial. Food Funct..

[215] Ferramosca A., Di Giacomo M., Zara V. (2017). Antioxidant dietary approach in treatment of fatty liver: New insights and updates. World J. Gastroenterol..

[216] Huang Y., Lang H., Chen K., Zhang Y., Gao Y., Ran L., Yi L., Mi M., Zhang Q. (2020). Resveratrol protects against nonalcoholic fatty liver disease by improving lipid metabolism and redox homeostasis via the PPARα pathway. Appl. Physiol. Nutr. Metab..

[217] Wang J., Zou Q., Suo Y., Tan X., Yuan T., Liu Z., Liu X. (2019). Lycopene ameliorates systemic inflammation-induced synaptic dysfunction via improving insulin resistance and mitochondrial dysfunction in the liver-brain axis. Food Funct..

[218] Lama A., Pirozzi C., Mollica M.P., Trinchese G., Di Guida F., Cavaliere G., Calignano A., Mattace Raso G., Berni Canani R., Meli R. (2017). Polyphenol-rich virgin olive oil reduces insulin resistance and liver inflammation and improves mitochondrial dysfunction in high-fat diet fed rats. Mol. Nutr. Food Res..

[219] Echeverría F., Valenzuela R., Bustamante A., Álvarez D., Ortiz M., Espinosa A., Illesca P., Gonzalez-Mañan D., Videla L.A. (2019). High-fat diet induces mouse liver steatosis with a concomitant decline in energy metabolism: Attenuation by eicosapentaenoic acid (EPA) or hydroxytyrosol (HT) supplementation and the additive effects upon EPA and HT co-administration. Food Funct..

[220] Badolati N., Masselli R., Sommella E., Sagliocchi S., Di Minno A., Salviati E., Campiglia P., Dentice M., Tenore G.C., Stornaiuolo M. (2020). The Hepatoprotective Effect of Taurisolo, a Nutraceutical Enriched in Resveratrol and Polyphenols, Involves Activation of Mitochondrial Metabolism in Mice Liver. Antioxidants.

[221] Sánchez-Calvo B., Cassina A., Mastrogiovanni M., Santos M., Trias E., Kelley E.E., Rubbo H., Trostchansky A. (2021). Olive oil-derived nitro-fatty acids: Protection of mitochondrial function in non-alcoholic fatty liver disease. J. Nutr. Biochem..

[222] Zapata J., Castro-Sepulveda M., Soto-Alarcon S., Alvarez D., Bustamante A., Villarroel G., Gallardo A., Garcia-Diaz D.F., Valenzuela R., Echeverria F. (2023). Targeting Mitochondria for the Prevention and Treatment of Nonalcoholic Fatty Liver Disease: Polyphenols as a Non-pharmacological Approach. Curr. Med. Chem..

[223] Kim M.B., Lee J., Lee J.Y. (2024). Targeting Mitochondrial Dysfunction for the Prevention and Treatment of Metabolic Disease by Bioactive Food Components. J. Lipid Atheroscler..

[224] Cao K., Xu J., Zou X., Li Y., Chen C., Zheng A., Li H., Li H., Szeto I.M., Shi Y. (2014). Hydroxytyrosol prevents diet-induced metabolic syndrome and attenuates mitochondrial abnormalities in obese mice. Free Radic. Biol. Med..

[225] Ortiz M., Soto-Alarcón S.A., Orellana P., Espinosa A., Campos C., López-Arana S., Rincón M.A., Illesca P., Valenzuela R., Videla L.A. (2020). Suppression of high-fat dietinduced obesity-associated liver mitochondrial dysfunction by docosahexaenoic acid and hydroxytyrosol coadministration. Dig. Liver Dis..

[226] Rafiei H., Omidian K., Bandy B. (2017). Comparison of dietary polyphenols for protection against molecular mechanisms underlying nonalcoholic fatty liver disease in a cell model of steatosis. Mol. Nutr. Food Res..

[227] Rafiei H., Omidian K., Bandy B. (2019). Dietary polyphenols protect against oleic acid-induced steatosis in an in vitro model of NAFLD by modulating lipid metabolism and improving mitochondrial function. Nutrients.

[228] Cheng K., Jia P., Ji S., Song Z., Zhang H., Zhang L., Wang T. (2021). Improvement of the hepatic lipid status in intrauterine growth retarded pigs by resveratrol is related to the inhibition of mitochondrial dysfunction, oxidative stress and inflammation. Food Funct..

[229] Davis J.M., Murphy E.A., Carmichael M.D., Davis B. (2009). Quercetin increases brain and muscle mitochondrial biogenesis and exercise tolerance. Am. J. Physiol. Regul. Integr. Comp. Physiol..

[230] Rayamajhi N., Kim S.K., Go H., Joe Y., Callaway Z., Kang J.G., Ryter S.W., Chung H.T. (2013). Quercetin induces mitochondrial biogenesis through activation of HO-1 in HepG2 cells. Oxida. Med. Cell. Longev..

[231] Ruiz M.J., Fernández M., Picó Y., Mañes J., Asensi M., Carda C., Asensio G., Estrela J.M. (2009). Dietary administration of high doses of pterostilbene and quercetin to mice is not toxic. J. Agric. Food Chem..

[232] Kuo J.J., Chang H.H., Tsai T.H., Lee T.Y. (2012). Positive effect of curcumin on inflammation and mitochondrial dysfunction in obese mice with liver steatosis. Int. J. Mol. Med..

[233] Soto-Urquieta M.G., López-Briones S., Pérez-Vázquez V., Saavedra-Molina A., González-Hernández G.A., Ramírez-Emiliano J. (2014). Curcumin restores mitochondrial functions and decreases lipid peroxidation in liver and kidneys of diabetic *db/db* mice. Biol. Res..

[234] Du S., Zhu X., Zhou N., Zheng W., Zhou W., Li X. (2022). Curcumin alleviates hepatic steatosis by improving mitochondrial function in postnatal overfed rats and fatty L02 cells through the SIRT3 pathway. Food Funct..

[235] Perez Ternero C., Werner C.M., Nickel A.G., Herrera M.D., Motilva M.J., Böhm M., Alvarez de Sotomayor M., Laufs U. (2017). Ferulic acid, a bioactive component of rice bran, improves oxidative stress and mitochondrial biogenesis and dynamics in mice and in human mononuclear cells. J. Nutr. Biochem..

[236] Mollica M.P., Mattace Raso G., Cavaliere G., Trinchese G., De Filippo C., Aceto S., Prisco M., Pirozzi C., Di Guida F., Lama A. (2017). Butyrate Regulates Liver Mitochondrial Function, Efficiency, and Dynamics in Insulin-Resistant Obese Mice. Diabetes.

[237] Yu X., Xu Y., Zhang S., Sun J., Liu P., Xiao L., Tang Y., Liu L., Yao P. (2016). Quercetin Attenuates Chronic Ethanol-Induced Hepatic Mitochondrial Damage through Enhanced Mitophagy. Nutrients.

[238] Liu P., Lin H., Xu Y., Zhou F., Wang J., Liu J., Zhu X., Guo X., Tang Y., Yao P. (2018). Frataxin-Mediated PINK1-Parkin-Dependent Mitophagy in Hepatic Steatosis: The Protective Effects of Quercetin. Mol. Nutr. Food Res..

[239] Cao P., Wang Y., Zhang C., Sullivan M.A., Chen W., Jing X., Yu H., Li F., Wang Q., Zhou Z. (2023). Quercetin ameliorates nonalcoholic fatty liver disease (NAFLD) via the promotion of AMPK-mediated hepatic mitophagy. J. Nutr. Biochem..

[240] Zhu L., Cao F., Hu Z., Zhou Y., Guo T., Yan S., Xie Q., Xia X., Yuan H., Li G. (2024). Cyanidin-3-O-Glucoside Alleviates Alcoholic Liver Injury via Modulating Gut Microbiota and Metabolites in Mice. Nutrients.

[241] Dong Y., Yu M., Wu Y., Xia T., Wang L., Song K., Zhang C., Lu K., Rahimnejad S. (2022). Hydroxytyrosol Promotes the Mitochondrial Function through Activating Mitophagy. Antioxidants.

[242] Jung S., Bae H., Song W.S., Jang C. (2022). Dietary Fructose and Fructose-Induced Pathologies. Annu. Rev. Nutr..

[243] Alami F., Alizadeh M., Shateri K. (2022). The effect of a fruit-rich diet on liver biomarkers, insulin resistance, and lipid profile in patients with non-alcoholic fatty liver disease: A randomized clinical trial. Scand. J. Gastroenterol..

[244] Du L.J., He Z.Y., Gu X., Hu X., Zhang X.X., Yang L.J., Li J., Pan L.Y., Li Y.Q., Yang B. (2022). Inverse Association of Fruit and Vegetable Consumption with Nonalcoholic Fatty Liver Disease in Chinese Patients with Type 2 Diabetes Mellitus. Nutrients.

[245] Wang R., Yan R., Jiao J., Li F., Zhang H., Chang Z., Wei H., Yan S., Li J. (2024). Fruit and vegetable intake and the risk of non-alcoholic fatty liver disease: A meta-analysis of observational studies. Front. Nutr..

[246] Notarnicola M., Tutino V., De Nunzio V., Cisternino A.M., Cofano M., Donghia R., Giannuzzi V., Zappimbulso M., Milella R.A., Giannelli G. (2024). Daily Orange Consumption Reduces Hepatic Steatosis Prevalence in Patients with Metabolic Dysfunction-Associated Steatotic Liver Disease: Exploratory Outcomes of a Randomized Clinical Trial. Nutrients.

[247] Sadeghi F., Amanat S., Bakhtiari M., Asadimehr H., Okhovat M.A., Hosseinzadeh M., Mazloomi S.M., Gholamalizadeh M., Doaei S. (2021). The effects of high fructose fruits and honey on the serum level of metabolic factors and nonalcoholic fatty liver disease. J. Diabetes Metab. Disord..

[248] Pathak M.P., Pathak K., Saikia R., Gogoi U., Patowary P., Chattopadhyay P., Das A. (2023). Therapeutic potential of bioactive phytoconstituents found in fruits in the treatment of non-alcoholic fatty liver disease: A comprehensive review. Heliyon.

[249] Sánchez-Terrón G., Martínez R., Delgado J., Molina J., Estévez M. (2024). Hepatoprotective mechanisms of pomegranate bioactives on a murine models affected by NAFLD as analysed by MS-based proteomics: The mitochondria in the eye of the storm. Food Res. Int..

[250] Crescenzo R., Bianco F., Mazzoli A., Giacco A., Liverini G., Iossa S. (2016). A possible link between hepatic mitochondrial dysfunction and diet-induced insulin resistance. Eur. J. Nutr..

[251] Cioffi F., Senese R., Lasala P., Ziello A., Mazzoli A., Crescenzo R., Liverini G., Lanni A., Goglia F., Iossa S. (2017). Fructose-Rich Diet Affects Mitochondrial DNA Damage and Repair in Rats. Nutrients.

[252] Swapna Sasi U.S., Sindhu G., Raghu K.G. (2020). Fructose-palmitate based high calorie induce steatosis in HepG2 cells via mitochondrial dysfunction: An in vitro approach. Toxicol. Vitr..

[253] Petito G., Giacco A., Cioffi F., Mazzoli A., Magnacca N., Iossa S., Goglia F., Senese R., Lanni A. (2023). Short-term fructose feeding alters tissue metabolic pathways by modulating microRNAs expression both in young and adult rats. Front. Cell Dev. Biol..

